# Effect of exercise intervention on depression in children and adolescents: a systematic review and network meta-analysis

**DOI:** 10.1186/s12889-023-16824-z

**Published:** 2023-10-04

**Authors:** Jiayu Li, Xianxian Zhou, Zan Huang, Tianyi Shao

**Affiliations:** College of Physical Education and Health Sciences, Zhejiang Normal University, Jinhua, 321004 Zhejiang China

**Keywords:** Exercise intervention, Child and adolescent, Depression, Network meta-analysis

## Abstract

**Objectives:**

To evaluate the effect of different exercise interventions on depressive symptoms in children and adolescents.

**Methods:**

Randomized controlled trials (RCT) published until May 2023 were screened in four databases. The Cochrane collaboration tool was used to assess the risk of bias for quality evaluation. Stata 16.0 software was used for both a pairwise meta-analysis and a series of frequentist network meta-analyses (NMA).

**Results:**

A total of 35 RCTs and 5393 participants were included. Aerobic exercise had the most significant effect on depressive symptoms (66.2%), followed by group training (62.5%), resistance exercise (59.0%), and aerobic combined with resistance exercise (57.9%). Furthermore, children and adolescents younger than 15 years showed significant improvement in depressive symptoms (SMD=-0.41, 95% CI (-0.63, -0.19), P < 0.01). The study also found a significant improvement in depression among healthy, obesity, and depressed populations (SMD=-0.25, 95% CI (-0.41, -0.08), P < 0.01); SMD=-0.15, 95% CI (-0.31, -0.00), P < 0.01; SMD=-0.75, 95% CI (-1.32, -0.19), P < 0.01). Additionally, 30 min of exercise had a significant effect (SMD=-0.14, 95% CI (-0,81, -0.01), P < 0.01), and 40–50 min of exercise had the best effect (SMD=-0.17, 95% CI (-0,33, -0.02), P < 0.01). Lastly, exercise frequency of three times per week was significant in children and adolescents (SMD=-0.42, 95% CI (-0,66, -0.18), P < 0.01).

**Conclusion:**

Exercise significantly improves depressive symptoms in children and adolescents, with aerobic exercise having the most significant effect. A 12-week, three-times-a-week, 40-50-minute exercise intervention was found to be more effective in younger children and adolescents.

**Supplementary Information:**

The online version contains supplementary material available at 10.1186/s12889-023-16824-z.

## Background

According to the World Health Organization (WHO), approximately 150 million people worldwide suffer from depression [1]. The prevalence of depression in children and adolescents is approximately 5–8% [2]. Children with depression are more likely to have deficits in cognitive development and underperform academically compared to normal children and adolescents [3–4]. They are also at increased risk of smoking, substance abuse, obesity, and suicide [5]. Psychotherapy, cognitive therapy, and medication have been widely used by educators and psychologists to improve depressive symptoms in children and adolescents, with some degree of success [6–8]. However, the disease burden of depression in children and adolescents has increased due to increased medication tolerance, widespread use of electronic devices, and decreased participation in outdoor activities [9].

In the past decades, exercise interventions have been used as an effective way to improve symptoms in depressed children and adolescents [10, 11]. Ample research has shown that exercise interventions can reduce depressive symptoms in children and adolescents [12–14]. Possible mechanisms of action include: (1) Exercise involvement have antidepressant effects by improving mood by releasing dopamine and endorphins [15]. (2) Exercise involvement reduces symptoms of stress and depression by decreasing cortisol, reducing symptoms of stress and depression [16]. (3) Exercise involvement promotes body metabolism and energy expenditure [17]. However, not all studies have confirmed the positive effect of exercise participation on improving depression in children and adolescents. Wang’s study showed that exercise interventions such as yoga were ineffective for depressive symptoms [18]. There is still controversy regarding the effect of exercise interventions on depressive symptoms in children and adolescents. Previous meta-analyses have weakened the interpretation of findings due to small sample sizes (21 studies) [19]. Furthermore, fewer studies have been conducted on exercise interventions for treating depression in children and adolescents compared to adults, particularly with respect to exploring exercise-related variables such as frequency and duration [20–22].

The purpose of this systematic review and network meta-analysis was to comprehensively analyze the effects of exercise interventions on improving depressive symptoms in children and adolescents and to assess differences in the effects of different exercise interventions (aerobic exercise, resistance exercise, aerobic combined with resistance exercise, group exercise). It provides a reference for research on the clinical medical treatment of depression and a theoretical basis for improving depressive symptoms in children and adolescents.

## Methods

### Protocol and registration

This review was performed according to Preferred Reporting Items for Systematic Reviews and Meta-Analysis (PRISMA) guidelines [23], and the Cochrane handbook for systematic reviews and meta-analysis [24]. The PRISSMA checklist is presented in Additional file 1. This meta-analysis was registered in PROSPERO (Number: CRD42023434355).

### Search strategy and information sources

A comprehensive search was done systematically through Scopus, PubMed, Web of Science, and APA PsycInfo up to the 30th of May 2023. Searching terms were based on adapted PICO questions to search through the aforementioned databases to accesses all the important articles. Free text words and medical subject heading (MeSH) terms were used. [1] (youth OR adolescent OR teenager OR child OR student OR boy OR girl); [2] (“physical activity” OR “physical exercise” OR “sports activities” OR “sport movement” OR sport* OR motor OR “athletic sports” OR “aerobic exercise " OR “aerobic training” OR “resistance exercise” OR “strength training” OR “muscle-strengthening exercise” OR “physical education” OR “fitness game”); [3] (depression OR depressive OR depressed OR melancholia OR dysphoria OR despair OR despondency OR “’ mental health” OR “emotional depression” OR “depressive symptom”). Details of the search strategy have been provided Additional file 2. Finally, the references of the included studies and relevant studies were manually searched by senior experts in the field to supplement the electronic literature database search for missing literature. The search was performed independently by two researchers (JYL and XXZ), and a third researcher (ZH) was consulted in case of disagreement.

### Eligibility criteria of the selected studies

The inclusion criteria for articles were determined using the PICOS (Participants/Interventions/Comparisons/Outcomes/Study Design) principles, as follows. Participants (P): children and adolescents who were diagnosed with depression or minor depression/dysthymia. Intervention (I): interventions in the form of exercise, aerobic exercise, (e.g., walking, running, cycling) and non-aerobic exercise (e.g., resistance exercise, strength exercise, weight lifting). Comparison (C): placebo treatment, usual care, psychotherapy, or no treatment. Outcome (O): a reduction in depressive symptoms or remission. Study Design (S): randomized controlled trials (RCTs).

Exclusion criteria included: (1) studies that included participants aged over 18 years; (2) studies in which the effects of exercise interventions could not be isolated because they were part of a multi-component intervention (e.g., diet and exercise interventions); (3) studies that did not provide details about the intensity of the exercise intervention; (4) studies that did not evaluate the outcomes of interest of the article(such as anxiety and stress); (5) studies that did not provide mean and standard deviation or data that could be translated into mean and standard deviation form; (6) non-peer-reviewed English, abstracts, reviews, conference; (7) only open access papers were included.

### Study selection

All references for the studies selected for this review were managed in EndNoteX9. After removing duplicates, the screening was conducted by separate researchers (JYL, ZH). The researchers screened the remaining studies for eligibility by reviewing study titles and abstracts, then the full-text reports (JYL, XXZ) to evaluate their appropriateness to be included in the systematic review. For these steps, agreement was reached between two researchers and disagreement was resolved by consensus with a third researcher (TYS).

### Data extraction

Two researchers separately performed data extraction (ZH, XXZ). The extracted data were compared for all included studies, and disagreement was solved with a third reviewer (JYL). The extracted data included the first author’s name, year of publication, basic information about the study population (number, age range, country, and region), depression measures, type of intervention, and outcome indicators (Additional file 3). The primary outcome indicator of the study involved depression emotions in children.

### Assessment of quality of individual studies

Two authors (JYL and ZH) independently assessed the risk of bias. The Cochrane Collaboration risk of bias instrument was used to assess bias across seven categories [25]: Random sequence generation (selection bias); Allocation concealment (selection bias); blinding of participants and personnel (performance bias);Blinding of outcome assessment (detection bias); Incomplete outcome data (attrition bias); Selective reporting (reporting bias); Other bias. As recommended, we rated each item as: 1) “little risk of bias” if it is completely fulfilled quality standards with the least bias; 2) “unclear” if it is plausible that a bias raises some doubt about the results; and 3) “high risk of bias” if it is plausible that a bias seriously weakens confidence in the results. In cases of disagreement, the rest of the group was consulted, and judgment was made based on consensus.

### Summary measures

Stata 16.0 software was used for pairwise meta-analysis and network meta-analysis. The methodological quality of the literature was evaluated using Review Manager 5.3 software, and a schematic diagram for risk of bias assessment was drawn. Effect sizes were combined using standardized mean difference (SMD) and 95% confidence interval (CI) for statistics.

### Synthesis of results

The pairwise meta-analysis was preceded by a heterogeneity test, using *I*^*2*^ to test for heterogeneity among included studies. If *I*^*2*^ ≤ 50%, it means that statistical heterogeneity among the study results is small, and the fixed effects model is selected for meta-analysis; if *I*^*2*^ > 50%, it means that there is significant statistical heterogeneity among the study results, and the randomized effects model is used [26].

The network meta-analysis first drew a reticulated evidence map to visualize the relationship between direct and indirect comparisons among exercise interventions. When there was a closed-loop structure, the Loop Inconsistency Test (LIT) was performed to check the inconsistency of the closed-loop structure, and the inconsistency factors (IF) and 95% CI and significant P values were calculated [27]. If P > 0.05, IF value < 1, and 95% CI’s contain 0, the consistency of each closed loop is good; otherwise, inconsistency is considered to exist [28]. Intervention effects were ranked using surface under the cumulative ranking curve (SUCRA), with SUCRA values ranging from 0 to 100%; the closer to 100%, the better the intervention will be.

### Publication bias

Funnel plots were drawn and Begg’s and Egger’s tests were used to detect whether the literature had publication bias.

## Results

### Search results and overview

**Fig. 1:**
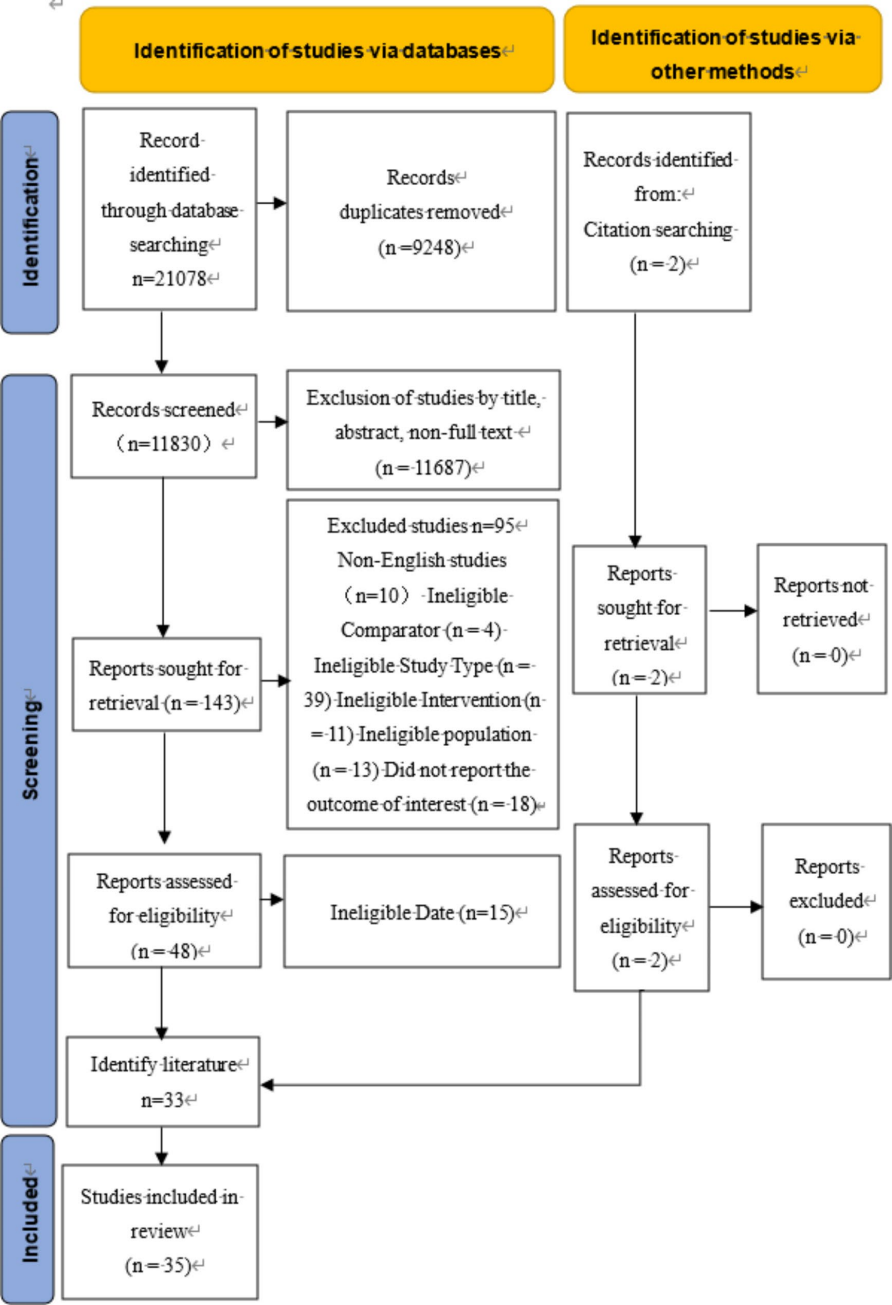
Flow chart of literature retrieval

A total of 21,078 studies were retrieved after systematically searching individual databases. After removing duplicate literature, 11,830 studies remained. After screening the titles and abstracts, 143 studies were read in full text for refinement (Additional file 4). After that, 33 studies met the inclusion and exclusion criteria. A manual search of references of included literature and relevant studies by senior experts in the field added two additional relevant pieces of literature. The final 35 studies were included for meta-analysis (Fig. 1).

### Study characteristics

The meta-analysis included thirty-five studies published between 1982 and 2023, with a total of 5,393 participants and a mean age of 13.23 ± 3.12 years. The studies were conducted in ten countries, with fifteen in the United States, four in the United Kingdom, three in Australia, China, and Iran, two in Germany, and one each in Israel, Mexico, Korea, Brazil, and Canada. The exercise interventions were classified as follows: aerobic exercise (AE), which involved continuous or low-intensity intermittent exercises such as running; resistance training (RT), which involved resistance exercises performed by overcoming self-body mass or applying external resistance; group training (GT), which involved team sports such as soccer, basketball, and physical activity; and medium-low-intensity multi-motion training (MT), which involved martial arts, yoga, aerobics, or aerobics combined with resistance exercise. Of the thirty-five studies, twenty involved AE, sixteen involved MT, five involved GT, two involved RT, and thirty- five involved controls who did not receive the exercise intervention. The mean intervention period was 13.94 ± 7.03 weeks, with 57% of the studies having an intervention period of greater than or equal to 12 weeks. The mean number of interventions per week was 3.35 ± 1.73, and the mean duration of each intervention was 45.3 ± 18.72 min.

### Outcome measures

The primary outcome was assessed using a validated depression rating scale to measure depressive symptoms at the end of the intervention period and the last available follow-up visit. If multiple scales were used, observer-rated scales were given priority over self-reported scales. The most commonly used scale was selected when multiple depression scales were reported to reduce heterogeneity. The specific characteristics of the included studies are described in Additional file 5.

### Quality appraisal

**Fig. 2:**
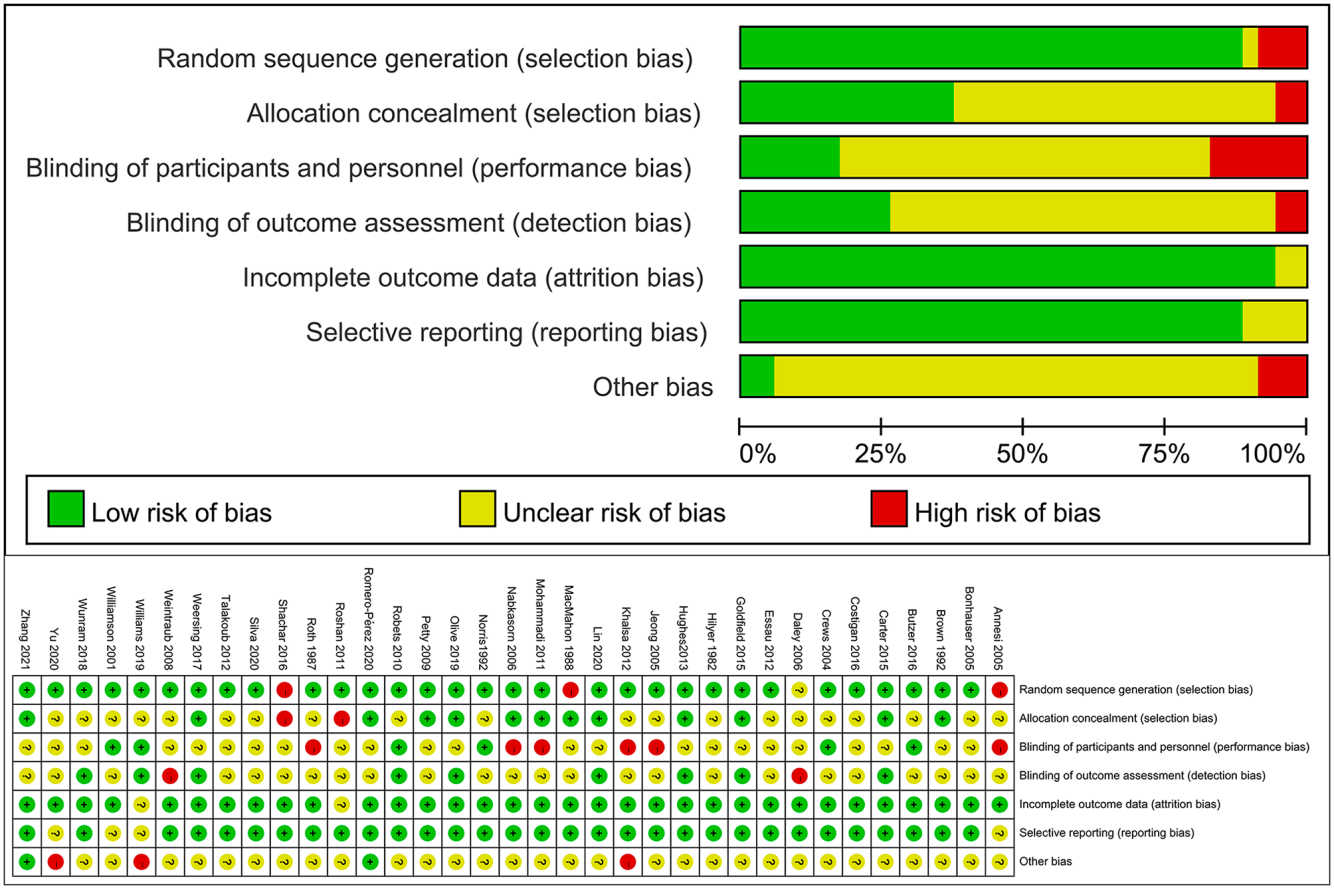
Graph of Cochrane risk bias assessment

The quality evaluation results of 35 articles are shown in Fig. 2. Among them, 31 articles explain the specific method of random grouping, 13 articles are hidden by allocation, 6 articles are blind by participants, 8 articles are blind by assessors, 33 articles are data integrity, 31 articles are selective reports and 2 articles are other biases (Additional file 7). The highest risk of bias stems from a lack of blinding participants. Concerning the remaining risk of bias indicators, a lack of information was observed in many studies. Therefore, the actual risk for the presence of biases in the included studies remains mostly unknown.

### Pairwise meta-analyses

The results of the pairwise meta-analysis show a significant improvement in depression among children and adolescents who engaged in exercise (Table 1). A summary forest plot of all exercise interventions is provided in Additional file 6. Subgroup analyses based on different exercise modalities revealed that AE [SMD=-0.36, 95% CI (-0.59, -0.12), P < 0.01], and aerobic combined with resistance exercise (MT) had a significant effect on children and adolescent depression [SMD=-0.24, 95% CI (-0.45, -0.03), P < 0.01], while RT and GT had a non-significant effect (P > 0.05). Age-wise, significant improvement was observed in children and adolescents under 15 years of age [SMD=-0.41, 95% CI (-0.63, -0.19), P < 0.01]. The effect was significant for depressed patients [SMD=-0.75, 95% CI (-1.32, -0.19), P < 0.01], healthy individuals [SMD=-0.25, 95% CI (-0.41, -0.08), P < 0.01], and obese individuals [SMD=-0.15, 95% CI (-0.31, -0.00), P < 0.01], with the best effect observed in some psychiatric patients. The effect of 12 weeks of intervention was significant [SMD=-0.38, 95% CI (-0.56, -0.19), P < 0.01], while the effect of 6 consecutive weeks of intervention was not significant. In terms of the duration of exercise sessions, the effect of 30 min of exercise was significant [SMD=-0.14, 95% CI (-0.81, -0.01), P < 0.01], while 60 min of exercise was not significant (P > 0.05). The best effect was observed with 40–50 min of exercise [SMD=-0.17, 95% CI (-0.33, -0.02), P < 0.01]. Regarding exercise frequency, the effect of three times per week was significant in children and adolescents [SMD=-0.42, 95% CI (-0.66, -0.18), P < 0.01], while the effect of two times per week or greater than four times per week was not significant (P > 0.05).

**Table 1:** Results of pairwise meta-analyses

Variable		Number of comparisons	Heterogeneity test results		Meta-analysis results	
			Q value	I^2^ value	SMD effect size (95% CI)	P value
Type	AE	20	78.14	77.0%	-0.36(-0.59, -0.12)	<0.01
	MT	14	68.12	80.9%	-0.24(-0.45, -0.03)	<0.01
	RT	2	0.19	0.0%	-0.08(-0.36, 0.20)	= 0.557
	GT	5	51.80	92.3%	-0.26(-0.71, 0.18)	= 0.240
Age, y	<15	17	147.76	89.2%	-0.41(-0.63, -0.19)	P<0.01
	≥ 15	11	29.66	66.3%	-0.24(-0.49, 0.01)	P = 0.062
Healthy status	Healthy	19	85.89	77.9%	-0.25(-0.41, -0.08)	P<0.01
	Obesity	6	2.94	0.0%	-0.15(-0.31, -0.00)	P<0.05
	Mental illness	9	89.84	91.1%	-0.75(-1.32, -0.19)	P<0.01
Session duration, min	30	7	30.92	77.4%	-0.14(-0.81, -0.01)	P<0.05
	40–50	13	35.24	63.1%	-0.17(-0,33, -0.02)	P<0.05
	60	7	15.19	60.5%	-0.13(-0.33, 0.07)	P = 0.200
Program duration, wk	6	5	23.32	82.8%	-0.37(-0.86, 0.12)	P = 0.137
	8	5	38.76	89.7%	-0.65(-1.49, 0.18)	P = 0.124
	12	6	6.23	19.7%	-0.38(-0.56, -0.19)	P<0.01
	>12	14	110.62	88.2%	-0.23(-0.46, -0.00)	P<0.05
Frequency, d/wk	2	6	39.77	87.4%	-0.41(-0.85, 0.03)	P = 0.06
	3	16	51.45	70.8%	-0.42(-0.66, -0.18)	P<0.01
	≥ 4	8	20.85	66.4%	-0.19(-0.40, 0.02)	P = 0.08
overall					-0.28(-0.41, -0.14)	P<0.01

### Network meta-analysis

**Fig. 3:**
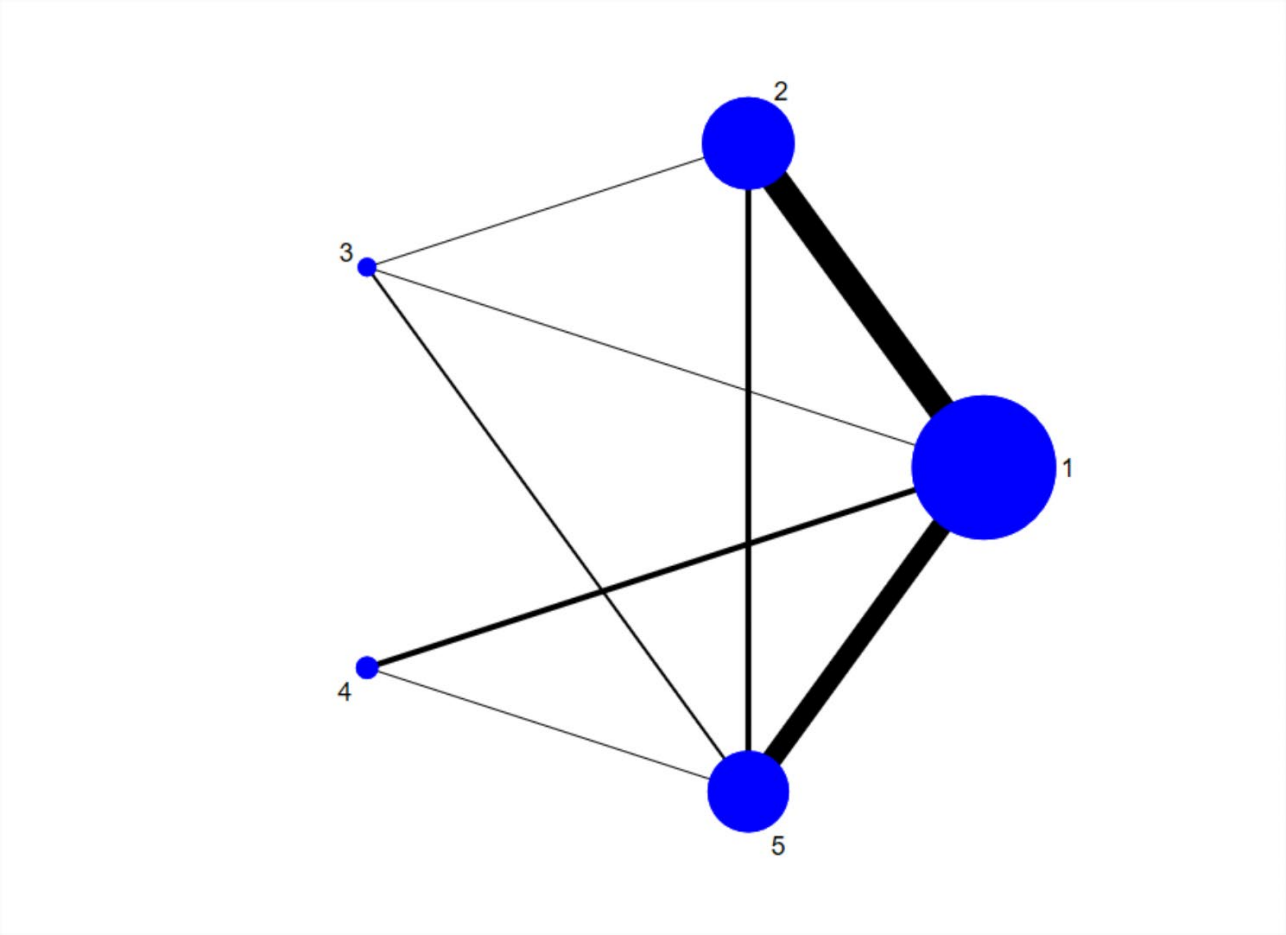
Geometry of the network. The size of the node represents the number of participants in each intervention. The thickness of the edges represents the number of studies in each treatment comparison. 1 = control; 2 = aerobic exercise; 3 = resistance training; 4 = group training; 5 = aerobics combined with resistance exercise

Based on the pairwise meta-analysis, this study included 35 studies with 5293 participants in a network meta-analysis. The dots in Fig. 3 represent different exercise interventions, with the dot’s size indicating the sample size, and the lines between the dots indicating a direct comparison between the two exercises. The thickness of the line represents the number of studies that made the comparison. If there is no line between two interventions, they cannot be directly compared and require reticulated meta-analysis. The overall inconsistency test showed that the P-values were all greater than 0.05 for all outcome indicators, indicating good overall consistency (Additional file 8). Further examination of the consistency of each closure showed that the inconsistency factor (IF) values ranged from 0.06 to 1.06, and the lower limits of 95% CI all contained 0, indicating good consistency for each closure. Thus, the consistency model was used for analysis.

### Network meta-analysis and probability ranking

**Fig. 4:**
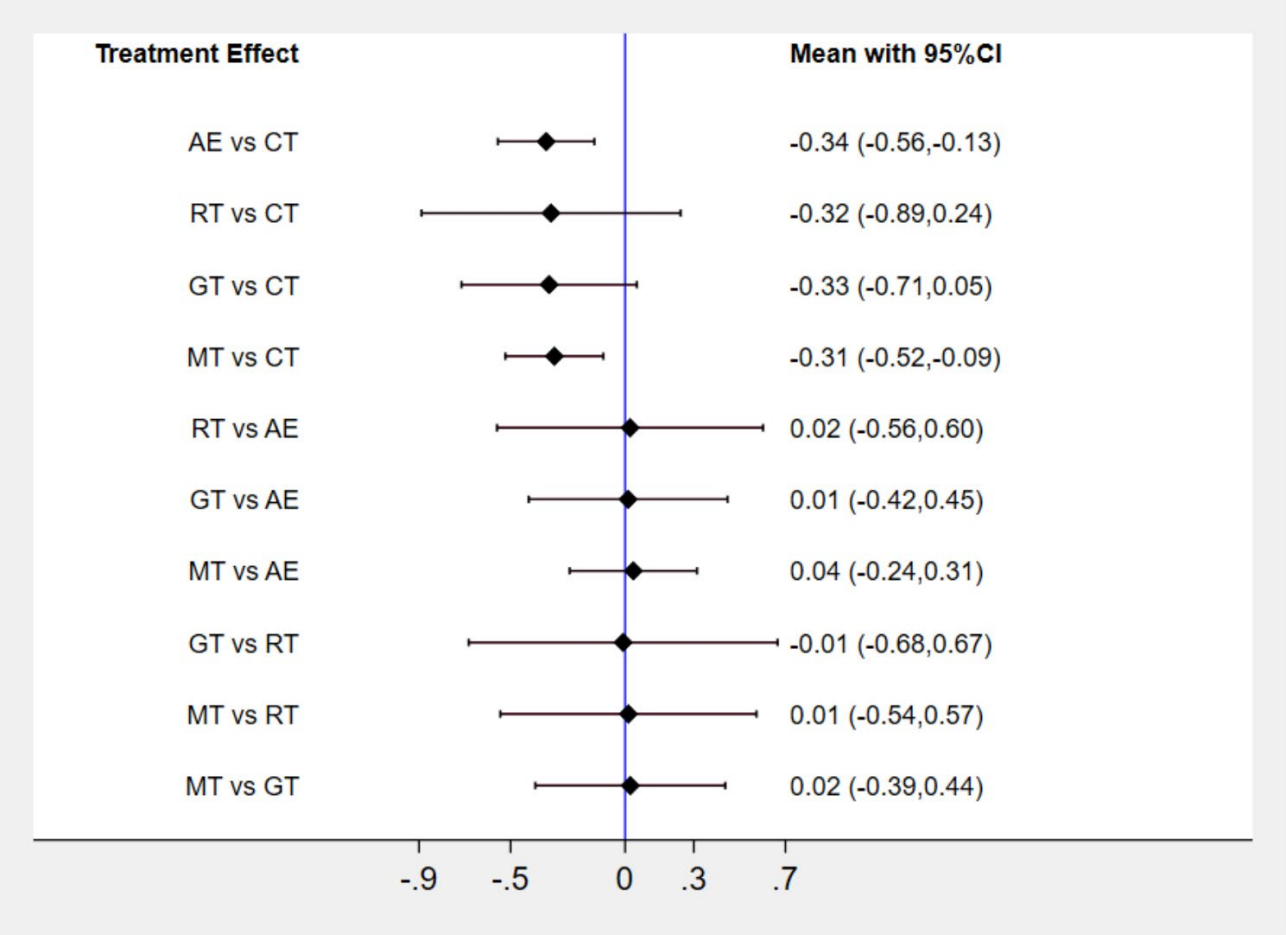
Results of network meta-analysis-Interval plot

The results of the network meta-analysis demonstrated that AE, MT, RT and GT significantly reduced depressed mood in children and adolescents compared to the control group (P < 0.01) (Fig. 4). According to the effect size and probability ranking scale, the SUCRA values for the intervention effects of the four types of exercise were ranked as follows, in descending order: AE (66.2%), GT (62.5%), RT (59.0%), and MT (57.9%). (Table 2)

**Table 2:** Ranking table of sport’s type

Treatment	SUCRA	PrBest	Mean Rank
CT	4.4	0.0%	4.8
AE	66.2	23.8%	2.4
RT	59.0	33.9%	2.6
GT	62.5	30.0%	2.5
MT	57.9	12.3%	2.7

### Sensitivity analysis

**Fig. 5:**
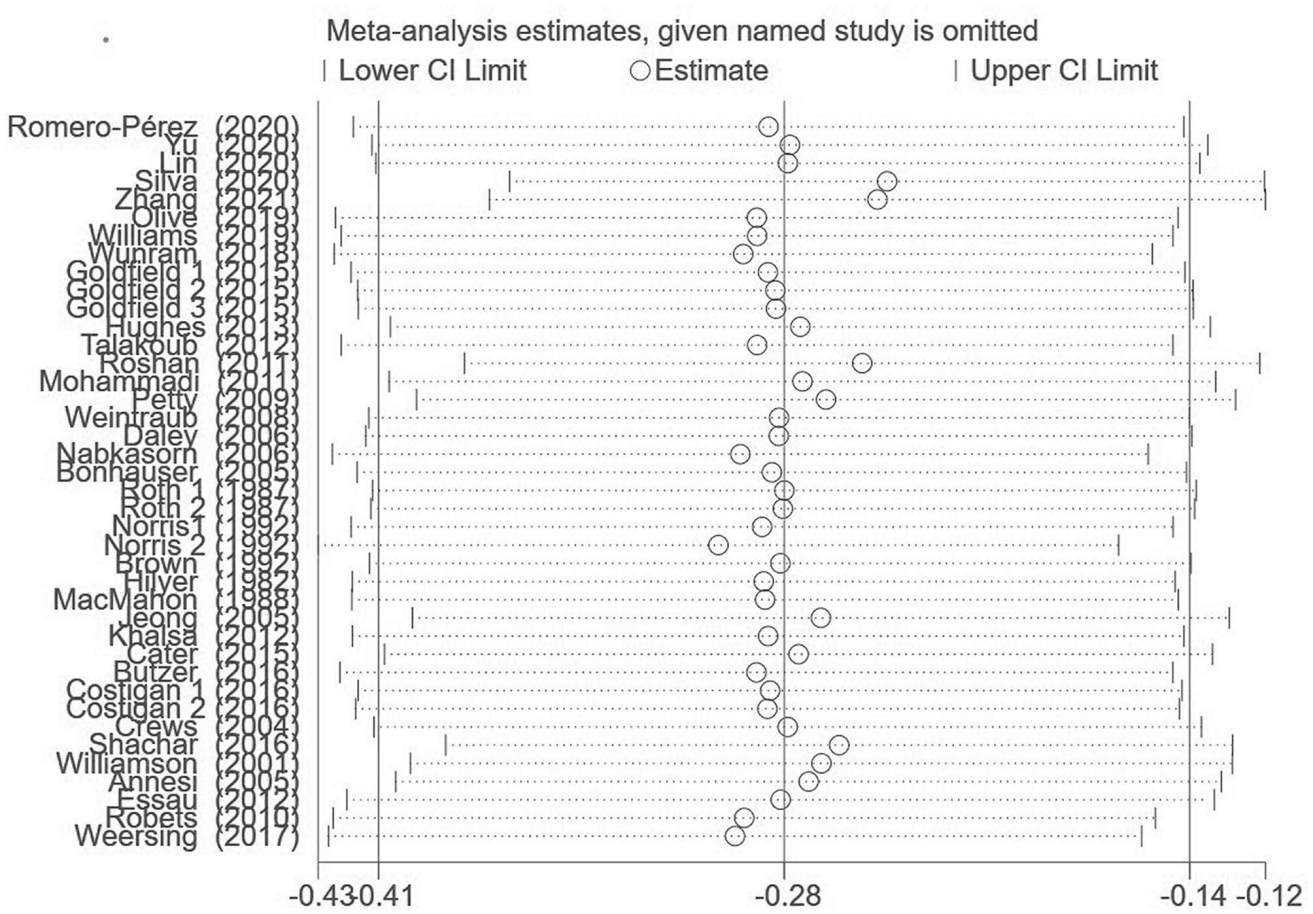
Sensitivity analysis of studies on the relationship between children’s participation in sports and negative emotions

In order to explore the sources of heterogeneity, sensitivity analyses were conducted by excluding individual studies in turn, and the results are presented in Fig. 5. Consistent with the initial analysis results, the exclusion of single studies had little effect on the combined results, indicating that the results of the combined effect values of this study were more stable.

### Publication bias test

**Fig. 6:**
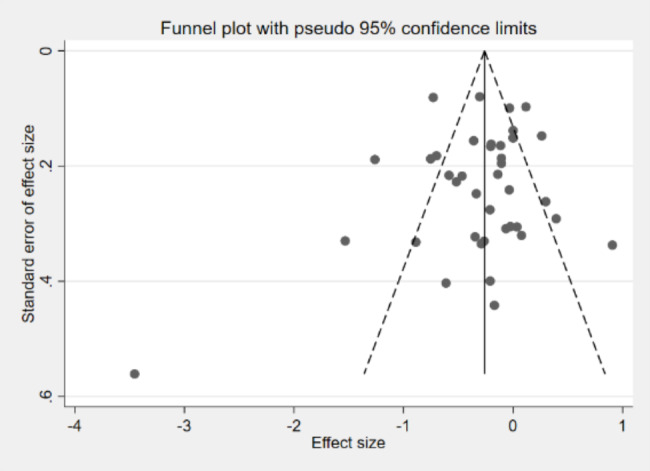
Funnel plot of publication bias for studies related to the relationship between children’s participation in motor activities and negative emotions

In addition, a publication bias test was performed on the included literature, and the results are shown in Fig. 6. An asymmetry at the bottom right of the funnel plot suggests the possibility of publication bias, but the method is subject to subjective judgment and may be inaccurate. Therefore, Begg’s and Egger’s tests were also applied, and the results indicated that P > 0.05, indicating no significant publication bias in the literature.

## Discussion

In this study, we employed a network meta-analysis to evaluate the effects of different exercise modalities on depressed mood in children and adolescents, and we ranked the probabilities for each indicator. Our results demonstrated that exercise significantly improved depressive symptoms in children and adolescents, with AE being the most effective, followed by GT, then RT, and finally MT. These findings suggest that various exercise interventions do not have an equal impact on improving depressive symptoms. AE is the primary component of most physical activities and can be easily incorporated into physical education classes. The AE process facilitates the release of hormones such as adrenaline and noradrenaline, which increase the body’s energy level and enhance its arousal state [29]. Depressed patients often have cardiovascular diseases, which can be a leading cause of death among them [30]. Therefore, AE can improve executive function [31] and reduce the risk of cardiovascular diseases in depressed patients [32]. GT are diverse, easy to maintain interest, and have high compliance among children and adolescents. They provide a platform for social interaction, which can reduce loneliness and isolation, increase social support, and allow children and adolescents with depression to communicate and share their experiences and feelings with others [33, 34]. Previous studies have shown that GT help children and adolescents to have positive experiences and emotions, enhancing their self-identity, and contributing to a sense of joy in their lives [35–37]. This is consistent with the findings of the present study, which showed that GT can reduce depressive symptoms in child adolescents. A meta-analysis of young people aged 12–18 years found insignificant effects of exercises such as yoga and aerobics [38], which is consistent with our study’s results. The reason for this may be related to the fact that depressed patients often lack confidence and have a negative evaluation of their physical form and abilities [39]. They may feel anxious and uneasy about participating in group exercises as they are overly concerned about performing poorly in groups [40]. The improvement of depressive symptoms in children and adolescents through MT may be related to the promotion of body metabolism and energy expenditure [17]. Furthermore, it has been shown that MT can promote a sense of well-being and pleasure by releasing neurotransmitters such as endorphins and dopamine in the body [41]. Single RT may also be beneficial in treating depressive symptoms compared to MT, but further research is necessary to confirm the specific effects [42]. For individuals with depression, exercising under medical supervision is recommended to ensure safety and effectiveness. In general, four perspectives help explain the improvement of depression in children and adolescents through exercise interventions: (1) the distraction perspective suggests that exercise can distract children and adolescents from unfavourable stimuli and lead to significant improvements in depressed mood during and after the activity [43]. (2) the self-efficacy perspective suggests that exercise can be viewed as a challenging activity, and being successful in it can increase self-confidence and help resist depressive mood [44]. (3) the social interaction perspective suggests that the presence of social relationships inherent in exercise and the mutual support between individuals involved in exercise play an essential role in the impact of depressive mood [45]. and (4) the physiological perspective suggests that exercise participation increases synaptic transmission of monoamines and activates the secretion of endorphins [46, 47]. The inhibitory effect of these substances on the central nervous system, reducing pain and enhancing the active state of the brain, could be responsible for improving depressive mood after exercise [48].

The duration and frequency of exercise interventions may moderate their effects. In the present study, an exercise intervention of 40–50 min per day, 2–3 times per week, was found to be most effective in improving negative mood in children. Clinical guidelines recommend 45 min of moderate-intensity exercise, 3 days per week, to improve negative mood [49], which aligns with our study’s results. Our study also found that a 30-minute exercise session was beneficial, supporting the idea of a positive dose-response relationship between exercise duration/frequency and improvement in depressive symptoms [50]. However, the Canadian government’s general health promotion guidelines and the American Academy of Pediatrics recommend at least 60 min of moderate to vigorous exercise to maintain physical and mental health [51]. Some studies suggest that excessively prolonged exercise may trigger the effects of pro-protein anabolic androgenic steroids (Androgen of Anabolic Steroids), leading to a significant increase in irritability and aggression and potentially triggering negative emotions [52].

Regarding the effect of the length of exercise interventions on depressed mood in children and adolescents, the present study found that a 12-week exercise intervention was the most effective. This finding is supported by Baker’s study, which reported a significant reduction in depression in subjects who participated in 12 weeks of exercise through a combined analysis of multiple exercise programs [53]. Our findings are also supported by Trivedi et al.‘s study [54]. The hippocampus, the most persistently affected area of the brain in depressed patients, decreases in volume by approximately 5% [55]. Studies have found that exercise lasting more than 6 weeks can increase the volume of the left and right parts of the hippocampus and cortical regions in subjects [56]. An aerobic exercise intervention lasting up to 12 months increased hippocampal volume by approximately 2%, but after cessation of training, subjects’ hippocampal volume returned to baseline levels [57]. Therefore, long-term adherence to exercise is required to alleviate depression by increasing hippocampal volume. The Blumenthal study further corroborates that aerobic exercise lasting more than 12 weeks is as effective as pharmacological treatment for depressive symptoms [58]. However, it is unclear whether there are differences in the effects of exercise cycles on children’s negative emotions. Further studies with larger samples are still needed to verify their effectiveness due to the small number of included studies, which may affect the statistics of effect sizes.

Recchia’s meta-analysis explored the effect of physical activity on depressed mood in children and adolescents, showing that the intervention significantly reduced negative mood in children and adolescents under 13 years of age [59]. This supports our study’s findings that physical activity is a significant intervention for children and adolescents under the age of 15. A recent cohort study reported that between the ages of 12 and 16 years, children’s physical activity levels began to decline, while increased sedentary time was associated with more depressive symptoms [60]. Moreover, previous meta-analyses covered a wide age range of participants (12–25 years) [61]. which may have affected the summary results considering the physical and psychological differences between children and adolescents. A meta-analysis of exercise for treating depression in children and adolescents showed a small to moderate effect [62], but due to the small sample size of the studies, the authors indicated insufficient evidence to demonstrate the benefits of exercise. In contrast, our study utilized indirect comparative data, expanding the sample size and producing more stable results.

### Limitations

This study has several limitations. Firstly, while a comprehensive and systematic literature search was conducted, it is possible that studies using different keywords or published in other languages were not included. Secondly, the quality assessment of the included studies revealed missing information on blinding of participants and assessors, exercise intensity, and a lack of uniform criteria and controls for exercise intensity variables. Future studies should include more rigorous reporting of study design and characteristics to analyze potential moderators of treatment effects. Finally, all articles used self-reported outcomes, which may have influenced the overall results.

## Conclusions

The effects of exercise interventions on depressive symptoms in children and adolescents are related to the exercise modality; therefore, selecting appropriate and effective exercise modalities is crucial in preventing or reducing depression in this population. Based on the available evidence, this study provides preliminary confirmation of the ranking of the effects of the four exercise interventions, with AE being superior to GT, which is superior to RT, which is superior to MT. Additionally, an exercise intervention of 40–50 min, three times a week, for 12 weeks, was found to be more effective in children and adolescents younger than 15. However, due to the limitations of the original study, the strength of evidence for this conclusion still needs to be further validated by high-quality randomized controlled trials to enhance its reliability and achieve effective prevention or reduction of depression in children and adolescents.

We have no known conflict of interest to disclose.

### Electronic supplementary material

Below is the link to the electronic supplementary material.

Additional file 1: PRISMA NMA checklist

Additional file 2: Search strategy

Additional file 3: The study characteristic

Additional file 4: List of the excluded studies after a full-text review

Additional file 5: Outcome measurement

Additional file 6: Forest plot

Additional file 7: Risk of bias

Additional file 8: Assessment of Inconsistency

Additional file 9: Abbreviations

## Data Availability

All extracted data used in this review has been reported in the text, figures and tables (including Appendices).
