# Supplementary material for: Effect of exercise intervention on depression in children and adolescents: a systematic review and network meta-analysis
Source: BMC Public Health. 2023 Oct 4;23:1918. doi: 10.1186/s12889-023-16824-z (PMC10552327; doi:10.1186/s12889-023-16824-z)
Supplement: Supplementary file 2 — Additional file 2: Search strategy [file 12889_2023_16824_MOESM2_ESM.docx]

**Additional file 2-Search strategy**

| **Databases** | **Keywords** |
| --- | --- |
| PubMed=4357 | ("youth"[Title/Abstract] OR "youths"[Title/Abstract] OR "adolescent"[Title/Abstract] OR "adolescents"[Title/Abstract] OR "teenager"[Title/Abstract] OR "teenagers"[Title/Abstract] OR "child"[Title/Abstract] OR "children"[Title/Abstract] OR "student"[Title/Abstract] OR "students"[Title/Abstract] OR "boy"[Title/Abstract] OR "girl"[Title/Abstract]) AND ("physical activity"[Title/Abstract] OR "physical exercise"[Title/Abstract] OR "sport movement"[Title/Abstract] OR "sport"[Title/Abstract] OR "motor"[Title/Abstract] OR "athletic sports"[Title/Abstract] OR "aerobic exercise"[Title/Abstract] OR "aerobic training"[Title/Abstract] OR "resistance exercise"[Title/Abstract] OR "strength training"[Title/Abstract] OR "muscle-strengthening exercise"[Title/Abstract] OR "physical education"[Title/Abstract] OR "fitness game"[Title/Abstract]) AND ("depression"[Title/Abstract] OR "depressive"[Title/Abstract] OR "depressed"[Title/Abstract] OR "melancholia"[Title/Abstract] OR "dysphoria"[Title/Abstract] OR "despair"[Title/Abstract] OR "despondency"[Title/Abstract] OR "mental health"[Title/Abstract] OR "emotional depression"[Title/Abstract] OR "depressive symptom"[Title/Abstract]) |
| Scopus=6348 | ( TITLE-ABS (youth OR adolescent OR teenager OR child OR children OR student OR boy OR girl) AND TITLE-ABS ("physical activity" OR "physical exercise" OR "sport movement" OR sport OR motor OR "athletic sports" OR "aerobic exercise" OR "aerobic training" OR "resistance exercise" OR "strength training" OR "muscle-strengthening exercise" OR "physical education" OR "fitness game") AND TITLE-ABS (depression OR depressive OR depressed OR melancholia OR dysphoria OR despair OR despondency OR "'mental health" OR "emotional depression" OR "depressive symptom" ) ) |
| WOS=7330 | ((AB=(youth OR youths OR adolescent OR adolescents OR teenager OR teenagers OR child OR children OR student OR students OR boy OR girl)) AND AB=(“physical activity” OR “physical exercise” OR “sport movement” OR sport OR motor OR “athletic sports” OR “aerobic exercise” OR “aerobic training” OR “resistance exercise” OR “strength training” OR “muscle-strengthening exercise” OR “physical education” OR “fitness game”)) AND TS=(depression OR depressive OR depressed OR melancholia OR dysphoria OR despair OR despondency OR “‘mental health” OR “emotional depression” OR “depressive symptom”) |
| PsycINFO=3048 | AB ( youth OR youths OR adolescent OR adolescents OR teenager OR teenagers OR child OR children OR student OR students OR boy OR girl ) AND AB ( “physical activity” OR “physical exercise” OR “sport movement” OR sport OR motor OR “athletic sports” OR “aerobic exercise” OR “aerobic training” OR “resistance exercise” OR “strength training” OR “muscle-strengthening exercise” OR “physical education” OR “fitness game” ) AND AB ( depression OR depressive OR depressed OR melancholia OR dysphoria OR despair OR despondency OR “‘mental health” OR “emotional depression” OR “depressive symptom” ) |
