# Supplementary material for: Effect of exercise intervention on depression in children and adolescents: a systematic review and network meta-analysis
Source: BMC Public Health. 2023 Oct 4;23:1918. doi: 10.1186/s12889-023-16824-z (PMC10552327; doi:10.1186/s12889-023-16824-z)
Supplement: Supplementary file 3 — Additional file 3: The study characteristic [file 12889_2023_16824_MOESM3_ESM.docx]

**Additional file 3 -The study characteristic**

| **Author / Year** | **Country** | **Sample size** | **Sample characteristics** | **Intervention/frequency** | **Period/week** |
| --- | --- | --- | --- | --- | --- |
| Annesi 2005[1] | United States of America | T:49 C:41 | 26 girls and 23 boys 10.5±0.9 years | MT,45 minutes three days a week | 12 |
| Bonhauser 2005[2] | United States of America | T:98 C:100 | 198 students 15 years | MT,90 minutes three times a week | 24 |
| Brown 1992[3] | United States of America | T:11 C:5 | 27 adolescents with mental disorders 15.6 years | AE, three times per week | 9 |
| Butzer 2016[4] | United States of America | T:119 C:94 | 211 students 12.64±0.33 years | GT,35 minutes twice a week | 24 |
| Carter 2015[5] | Australia | T:21 C:22 | 65 adolescents 15.8±0.6 years | AE, MT,8-10 minutes three times a week | 8 |
| Costigan 2016[6] | Britain | T:44 C:43 | 87 adolescents 16.8±0.6 years | AE, twice a week | 6 |
| Crews 2004[7] | United States of America | T:34 C:32 | 66 students in grade 4 | AE,20 minutes three times a week | 6 |
| Daley 2006[8] | Britain | T:28 C:25 | 81 obese children 11-16 years | MT, three times a week | 8 |
| Essau 2012[9] | Germany | T:302 C:306 | 638 children 10.91±0.89 years | MT,60 minutes ten times a week | 12 |
| Goldfield 2015[10] | Canada | T:75 C:78 C:75 C:76 | Obese adolescents 15.6±1.4 years | AE, RT, MT,45 minutes per week | 22 |
| Hilyer 1982[11] | United States of America | T:14 C:12 | 30 severely depressed adolescents 12-18 years | AE, 30 minutes three times a week | 12 |
| Hughes 2013[12] | United States of America | T:20 C:23 | 60 male adolescents 15-18 years | RT,90 minutes three times a week | 20 |
| Jeong 2005[13] | South Korea | T:20 C:20 | 40 junior high school students 16 years | AE,45 minutes three times a week | 12 |
| Khalsa 2012[14] | United States of America | T:74 C:47 | 121 students 16.8±0.6 years | MT,45 minutes two or three times a week | 11 |
| Lin 2020[15] | China | T:20 C:19 | Adolescents 12-14 years | AE,30 minutes of running four days a week | 12 |
| MacMahon 1988[16] | United States of America | T:32 C:37 | 69 male adolescents 14-18 years | AE,40 minutes three times a week | 12 |
| Mohammadi 2011[17] | Iran | T:40 C:40 | 100 depressed children | GT, 75 minutes three times a week | 8 |
| Nabkasorn 2006[18] | Britain | T:19 C:19 C:20 | 147 adolescents | AE, MT,30 minutes twice a week | 10 |
| Norris 1992[19] | Britain | T:21 C:28 | 49 female students 18.8±0.7 years | AE,50 minutes five times a week | 8 |
| Olive 2019[20] | Australia | T:217 C:189 | Healthy children 8±0.6 years | MT, 50 minutes twice a week | 208 |
| Petty 2009[21] | United States of America | T:63 C:59 | 207 children 7-11 years | AE, 20 or 40 minutes per day | 13 |
| Roberts 2010[22] | Mexico | T:54 C:51 | Obese children 10.02±0.79 years | AE, 50 minutes twice a week | 20 |
| Romero-Pérez 2020[23] | Iran | T:24 C:24 | 24 depressed adolescents 17.5±2.24 years | AE, three times a week | 6 |
| Roshan 2011[24] | United States of America | T:19 C:18 C:18 | 1051 students | AE, MT,30 minutes three times a week | 11 |
| Roth 1987[25] | Australia | T:237 C:191 | Seventh graders 11.99±0.33 years | GT,60 minutes once a week | 20 |
| Shachar 2016[26] | Israel | T:330 C:319 | 649 children in grades 3-6 | GT,120-180min per week | 24 |
| Silva 2020[27] | Brazil | T:18 C:15 | Children with ADHD 11-14 years | AE, twice a week | 8 |
| Talakoub 2012[28] | Iran | T:90 C:85 | 72 children with diabetes 17.43±2.09 years | AE, 60 minutes three times a week | 6 |
| Weersing 2017[29] | United States of America | T:95 C:90 | 185 depressed children 11.3±2.6 years | MT,45 minutes per week | 24 |
| Weintraub 2008[30] | United States of America | T:9 C:12 | Obese children 9.50±0.58 years | GT,75 minutes three times a week | 24 |
| Williams 2019[31] | United States of America | T:90 C:85 | Overweight children 9.7±0.9 years | AE,40 minutes per day | 56 |
| Williamson 2001[32] | United States of America | T:64 C:64 | 64 children 9 to 10 years | MT,30min |  |
| Wunram 2018[33] | Germany | T:41 C:23 | 64 depressed children 13-18 years | AE,30 minutes four days a week | 6 |
| Yu 2020[34] | China | T:99 C:72 | Obese children 9.8±0.7 years | MT, 40 minutes five times a week | 32 |
| Zhang 2021[35] | China | T:66 C:69 | Children with depression 14.3±1.8 years | MT, 30 minutes three times a week | 16 |

1. Annesi, J.J. Correlations of depression and total mood disturbance with physical activity and self-concept in preadolescents enrolled in an after-school exercise program. *Psychological reports* **2005**, *96*, 891-898, doi:10.2466/pr0.96.4.891-898.

2. Bonhauser, M.; Fernandez, G.; Püschel, K.; et al. Improving physical fitness and emotional well-being in adolescents of low socioeconomic status in Chile: results of a school-based controlled trial. *Health Promotion International* **2005**, *20*, 113-122, doi:10.1093/heapro/dah603.

3. Brown, H.E.; Pearson, N.; Braithwaite, R.E.; et al. Physical activity interventions and depression in children and adolescents : a systematic review and meta-analysis. *Sports Med* **2013**, *43*, 195-206, doi:10.1007/s40279-012-0015-8.

4. Butzer, B.; LoRusso, A.; Shin, S.H.; et al. Evaluation of Yoga for Preventing Adolescent Substance Use Risk Factors in a Middle School Setting: A Preliminary Group-Randomized Controlled Trial. *Journal of Youth and Adolescence* **2017**, *46*, 603-632, doi:10.1007/s10964-016-0513-3.

5. Carter, T.; Guo, B.; Turner, D.; et al. Preferred intensity exercise for adolescents receiving treatment for depression: a pragmatic randomised controlled trial. *Bmc Psychiatry* **2015**, *15*, doi:10.1186/s12888-015-0638-z.

6. Costigan, S.A.; Eather, N.; Plotnikoff, R.C.; et al. High-Intensity Interval Training for Cognitive and Mental Health in Adolescents. *Medicine & Science in Sports & Exercise* **2016**, *48*, 1985-1993, doi:10.1249/mss.0000000000000993.

7. Crews, D.J.; Lochbaum, M.R.; Landers, D.M. Aerobic physical activity effects on psychological well-being in low-income Hispanic children. *Perceptual and motor skills* **2004**, *98*, 319-324, doi:10.2466/pms.98.1.319-324.

8. Daley, A.J.; Copeland, R.J.; Wright, N.P.; et al. Exercise therapy as a treatment for psychopathologic conditions in obese and morbidly obese adolescents: a randomized, controlled trial. *Pediatrics* **2006**, *118*, 2126-2134, doi:10.1542/peds.2006-1285.

9. Essau, C.A.; Conradt, J.; Sasagawa, S.; et al. Prevention of Anxiety Symptoms in Children: Results From a Universal School-Based Trial. *Behavior Therapy* **2012**, *43*, 450-464, doi:10.1016/j.beth.2011.08.003.

10. Goldfield, G.S.; Kenny, G.P.; Alberga, A.S.; et al. Effects of aerobic training, resistance training, or both on psychological health in adolescents with obesity: The HEARTY randomized controlled trial. *Journal of consulting and clinical psychology* **2015**, *83*, 1123-1135, doi:10.1037/ccp0000038.

11. Hilyer, J.C.; Wilson, D.G.; Dillon, C.; et al. Physical fitness training and counseling as treatment for youthful offenders. **1982**, *29*, 292.

12. Hughes, C.W.; Barnes, S.; Barnes, C.; et al. Depressed Adolescents Treated with Exercise (DATE): A pilot randomized controlled trial to test feasibility and establish preliminary effect sizes. *Mental Health and Physical Activity* **2013**, *6*, 119-131, doi:10.1016/j.mhpa.2013.06.006.

13. Jeong, Y.-J.; Hong, S.-C.; Lee, M.S.; et al. Dance movement therapy improves emotional responses and modulates neurohormones in adolescents with mild depression. *The International journal of neuroscience* **2005**, *115*, 1711-1720, doi:10.1080/00207450590958574.

14. Khalsa, S.B.S.; Hickey-Schultz, L.; Cohen, D.; et al. Evaluation of the mental health benefits of yoga in a secondary school: a preliminary randomized controlled trial. *The journal of behavioral health services & research* **2012**, *39*, 80-90, doi:10.1007/s11414-011-9249-8.

15. Lin, K.; Stubbs, B.; Zou, W.; et al. Aerobic exercise impacts the anterior cingulate cortex in adolescents with subthreshold mood syndromes: a randomized controlled trial study. *Translational Psychiatry* **2020**, *10*, doi:10.1038/s41398-020-0840-8.

16. MacMahon, J.R.; Gross, R.T.J.A.J.o.D.o.C. Physical and psychological effects of aerobic exercise in delinquent adolescent males. **1988**, *142*, 1361-1366.

17. Mohammadi, M. A study and comparison of the effect of team sports (soccer and volleyball) and individual sports (table tennis and badminton) on depression among high school students. *Aust. J. Basic Appl. Sci* **2011**, *5*, 1005-1011.

18. Nabkasorn, C.; Miyai, N.; Sootmongkol, A.; et al. Effects of physical exercise on depression, neuroendocrine stress hormones and physiological fitness in adolescent females with depressive symptoms. *European journal of public health* **2006**, *16*, 179-184.

19. Norris, R.; Carroll, D.; Cochrane, R. The effects of physical activity and exercise training on psychological stress and well-being in an adolescent population. *Journal of psychosomatic research* **1992**, *36*, 55-65, doi:10.1016/0022-3999(92)90114-h.

20. Olive, L.S.; Byrne, D.; Cunningham, R.B.; et al. Can Physical Education Improve the Mental Health of Children? The LOOK Study Cluster-Randomized Controlled Trial. *Journal of Educational Psychology* **2019**, *111*, 1331-1340, doi:10.1037/edu0000338.

21. Petty, K.H.; Davis, C.L.; Tkacz, J.; et al. Exercise effects on depressive symptoms and self-worth in overweight children: a randomized controlled trial. *Journal of pediatric psychology* **2009**, *34*, 929-939.

22. Roberts, C.M.; Kane, R.; Bishop, B.; et al. The prevention of anxiety and depression in children from disadvantaged schools. *Behaviour Research and Therapy* **2010**, *48*, 68-73, doi:10.1016/j.brat.2009.09.002.

23. Romero-Pérez, E.M.; González-Bernal, J.J.; Soto-Cámara, R.; et al. Influence of a physical exercise program in the anxiety and depression in children with obesity. *International journal of environmental research and public health* **2020**, *17*, 4655.

24. Roshan, V.D.; Pourasghar, M.; Mohammadian, Z. The efficacy of intermittent walking in water on the rate of MHPG sulfate and the severity of depression. *Iranian Journal of Psychiatry and Behavioral Sciences* **2011**, *5*, 26-31.

25. Roth, D.L.; Holmes, D.S. Influence of aerobic exercise training and relaxation training on physical and psychologic health following stressful life events. *Psychosomatic Medicine* **1987**, *49*.

26. Shachar, K.; Ronen-Rosenbaum, T.; Rosenbaum, M.; et al. Reducing child aggression through sports intervention: The role of self-control skills and emotions. *Children and Youth Services Review* **2016**, *71*, 241-249, doi:10.1016/j.childyouth.2016.11.012.

27. Da Silva, L.A.; Doyenart, R.; Salvan, P.H.; et al. Swimming training improves mental health parameters, cognition and motor coordination in children with Attention Deficit Hyperactivity Disorder. *International Journal of Environmental Health Research* **2020**, *30*, 584-592, doi:10.1080/09603123.2019.1612041.

28. Sedigheh, T.; Sakineh, G.; Marzieh, H.; et al. Impact of exercise on affective responses in female adolescents with type I diabetes. **2012**.

29. Weersing, V.R.; Brent, D.A.; Rozenman, M.S.; et al. Brief Behavioral Therapy for Pediatric Anxiety and Depression in Primary Care A Randomized Clinical Trial. *Jama Psychiatry* **2017**, *74*, 571-578, doi:10.1001/jamapsychiatry.2017.0429.

30. Weintraub, D.L.; Tirumalai, E.C.; Haydel, K.F.; et al. Team sports for overweight children: The Stanford sports to prevent obesity randomized trial (SPORT). *Archives of pediatrics & adolescent medicine* **2008**, *162*, 232-237.

31. Williams, C.F.; Bustamante, E.E.; Waller, J.L.; et al. Exercise effects on quality of life, mood, and self-worth in overweight children: the SMART randomized controlled trial. *Translational behavioral medicine* **2019**, *9*, 451-459.

32. Williamson, D.; Dewey, A.; Steinberg, H. Mood change through physical exercise in nine- to ten-year-old children. *Perceptual and motor skills* **2001**, *93*, 311-316, doi:10.2466/pms.93.5.311-316.

33. Wunram, H.L.; Hamacher, S.; Hellmich, M.; et al. Whole body vibration added to treatment as usual is effective in adolescents with depression: a partly randomized, three-armed clinical trial in inpatients. **2018**, *27*, 645-662.

34. Yu, H.-j.; Li, F.; Hu, Y.-f.; et al. Improving the metabolic and mental health of children with obesity: a school-based nutrition education and physical activity intervention in Wuhan, China. *Nutrients* **2020**, *12*, 194.

35. Zhang, J.; Ji, W. Exercise intervention improves the quality of life, anxiety, and depression of adolescent depression patients. *Int J Clin Exp Med* **2021**, *14*, 1292-1300.
