# Supplementary material for: Effect of exercise intervention on depression in children and adolescents: a systematic review and network meta-analysis
Source: BMC Public Health. 2023 Oct 4;23:1918. doi: 10.1186/s12889-023-16824-z (PMC10552327; doi:10.1186/s12889-023-16824-z)
Supplement: Supplementary file 4 — Additional file 4: List of the excluded studies after a full-text review [file 12889_2023_16824_MOESM4_ESM.docx]

**Additional file -4 List of the excluded studies after a full-text review**

| **Author, year** | **Reasons of exclusion** |
| --- | --- |
| Fang2017[1] | Ineligible Study Type (n = 39) |
| Craig2018[2] | Ineligible Study Type |
| Archer2014[3] | Ineligible Study Type |
| Annesi2004[4] | Ineligible Study Type |
| Carballo-Fazanes2022[5] | Ineligible Study Type |
| Tse 2020 [6] | Ineligible Study Type |
| Recchia 2023[7] | Ineligible Study Type |
| Kataoka 2002[8] | Ineligible Study Type |
| Kandola2020[9] | Ineligible Study Type |
| Kaku2022[10] | Ineligible Study Type |
| Gordon2018[11] | Ineligible Study Type |
| Gilbert2021[12] | Ineligible Study Type |
| Fergusson2005[13] | Ineligible Study Type |
| Brunoni2017[14] | Ineligible Study Type |
| Baena-Extremera2016 [15] | Ineligible Study Type |
| Ahlen2015[16] | Ineligible Study Type |
| Hetrick2012[17] | Ineligible Study Type |
| Zhao2020[18] | Ineligible Study Type |
| Xu2020[19] | Ineligible Study Type |
| Werner-Seidler2017[20] | Ineligible Study Type |
| Wang2022[21] | Ineligible Study Type |
| Stanton2014[22] | Ineligible Study Type |
| Song 2021[23] | Ineligible Study Type |
| Sallis2000[24] | Ineligible Study Type |
| Ruotsalainen2015[25] | Ineligible Study Type |
| Rodriguez-Ayllon 2019[26] | Ineligible Study Type |
| Recchia 2022[27] | Ineligible Study Type |
| Rajkumar2020[28] | Ineligible Study Type |
| Oberste2020[29] | Ineligible Study Type |
| Melton2016[30] | Ineligible Study Type |
| Maughan2013[31] | Ineligible Study Type |
| MacQueen2016[32] | Ineligible Study Type |
| Kvam2016[33] | Ineligible Study Type |
| Kennedy2008[34] | Ineligible Study Type |
| Kandola2019[35] | Ineligible Study Type |
| Josefsson2014[36] | Ineligible Study Type |
| Johnstone2018[37] | Ineligible Study Type |
| James-Palmer2020[38] | Ineligible Study Type |
| Hoare2016[39] | Ineligible Study Type |
| 김용주, 2008[40] | Non-English studies（n=10） |
| 변정은2019[41] | Non-English studies |
| Sun2014[42] | Non-English studies |
| So2009[43] | Non-English studies |
| Pantoja Vallejo2019[44] | Non-English studies |
| Niederer2017[45] | Non-English studies |
| Lu 2017[46] | Non-English studies |
| López-Rodríguez 2012[47] | Non-English studies |
| Knechtle2004[48] | Non-English studies |
| Choi2017[49] | Non-English studies |
| Neville2021[50] | Ineligible Comparator (n = 4) |
| Lawrence2015[51] | Ineligible Comparator |
| Wang2023[52] | Ineligible Comparator |
| Osborn2020[53] | Ineligible Comparator |
| Xie 2021[54] | Ineligible Intervention (n = 11) |
| Weersing 2017[55] | Ineligible Intervention |
| Schmaal 2016[56] | Ineligible Intervention |
| Salazar 2014[57] | Ineligible Intervention |
| Ranney2021[58] | Ineligible Intervention |
| Pophillat 2016[59] | Ineligible Intervention |
| Pojednic 2016[60] | Ineligible Intervention |
| Johnson2020[61] | Ineligible Intervention |
| Drieberg2019[62] | Ineligible Intervention |
| Downs2013[63] | Ineligible Intervention |
| Bodicherla2021[64] | Ineligible Intervention |
| Young 2015[65] | Irrelevant population (n = 13) |
| Wieber 2022[66] | Irrelevant population |
| Wang 2022 [67] | Irrelevant population |
| Tiner 2021[68] | Irrelevant population |
| Seok-Min 2008[69] | Irrelevant population |
| Song2021[70] | Irrelevant population |
| Rooney2013 [71] | Irrelevant population |
| Kim2018[72] | Irrelevant population |
| Kekäläinen2018[73] | Irrelevant population |
| Kanning2020[74] | Irrelevant population |
| Gordon2010[75] | Irrelevant population |
| Chang2017[76] | Irrelevant population |
| Callaghan2011[77] | Irrelevant population |
| de Bruin 2016[78] | Did not report the outcome of interest (n = 18) |
| Cocca2020[79] | Did not report the outcome of interest |
| Essau2012[80] | Did not report the outcome of interest |
| Williamson 2001[81] | Did not report the outcome of interest |
| Wilczyńska 2022[82] | Did not report the outcome of interest |
| Whitney 2019 [83] | Did not report the outcome of interest |
| Vani 2021[84] | Did not report the outcome of interest |
| Tercedor 2017[85] | Did not report the outcome of interest |
| Tao 2022[86] | Did not report the outcome of interest |
| Soltani 2021 [87] | Did not report the outcome of interest |
| Shomaker 2019 [88] | Did not report the outcome of interest |
| Salaj 2022[89] | Did not report the outcome of interest |
| Ridgers 2010 [90] | Did not report the outcome of interest |
| Min2015[91] | Did not report the outcome of interest |
| Bohnert2013[92] | Did not report the outcome of interest |
| Bazzano2018[93] | Did not report the outcome of interest |
| Battaglia2018[94] | Did not report the outcome of interest |
| Barrett2001[95] | Did not report the outcome of interest |
| Wang 2022[96] | Ineligible Date (n = 15) |
| Telles2013 [97] | Ineligible Date |
| Stella 2005[98] | Ineligible Date |
| Standage 2013 [99] | Ineligible Date |
| Shannon 2018 [100] | Ineligible Date |
| Roberts 2010[101] | Ineligible Date |
| Ren2020 [102] | Ineligible Date |
| Rasing 2020[103] | Ineligible Date |
| Piek,2008[104] | Ineligible Date |
| Olive2021[105] | Ineligible Date |
| Knubben2007[106] | Ineligible Date |
| Knapen2015[107] | Ineligible Date |
| Kall2015[108] | Ineligible Date |
| Jouper2009[109] | Ineligible Date |
| Annesi2005[110] | Ineligible Date |

1. Fang, H.; Quan, M.; Zhou, T.; et al. Relationship between Physical Activity and Physical Fitness in Preschool Children: A Cross-Sectional Study. *BioMed research international* **2017**, *2017*, 9314026, doi:10.1155/2017/9314026.

2. Craig, F.; Lorenzo, A.; Lucarelli, E.; et al. Motor competency and social communication skills in preschool children with autism spectrum disorder. *Autism research : official journal of the International Society for Autism Research* **2018**, *11*, 893-902, doi:10.1002/aur.1939.

3. Archer, T.; Josefsson, T.; Lindwall, M. Effects of Physical Exercise on Depressive Symptoms and Biomarkers in Depression. *Cns & Neurological Disorders-Drug Targets* **2014**, *13*, 1640-1653.

4. Annesi, J.J. Relationship between self-efficacy and changes in rated tension and depression for 9- to 12-yr.-old children enrolled in a 12-wk. after-school physical activity program. *Perceptual and motor skills* **2004**, *99*, 191-194, doi:10.2466/pms.99.1.191-194.

5. Carballo-Fazanes, A.; Rodrigues, L.P.; Silva, R.; et al. The Developmental Trajectory of Motor Competence of Children That Lived the COVID-19 Confinement Period: A Four-Year Follow-Up Study in Portuguese Children. *Journal of functional morphology and kinesiology* **2022**, *7*, doi:10.3390/jfmk7030064.

6. Tse, A.C.Y.J.J.o.A.; Disorders, D. Brief Report: Impact of a Physical Exercise Intervention on Emotion Regulation and Behavioral Functioning in Children with Autism Spectrum Disorder. **2020**, *50*.

7. Recchia, F.; Bernal, J.D.; Fong, D.Y.; et al. Physical Activity Interventions to Alleviate Depressive Symptoms in Children and Adolescents: A Systematic Review and Meta-analysis. **2023**.

8. Kataoka, S.H.; Zhang, L.; Wells, K.B. Unmet need for mental health care among U.S. children: variation by ethnicity and insurance status. *The American journal of psychiatry* **2002**, *159*, 1548-1555, doi:10.1176/appi.ajp.159.9.1548.

9. Kandola, A.; Lewis, G.; Osborn, D.P.J.; et al. Depressive symptoms and objectively measured physical activity and sedentary behaviour throughout adolescence: a prospective cohort study. *Lancet Psychiatry* **2020**, *7*, 262-271, doi:10.1016/s2215-0366(20)30034-1.

10. Kaku, S.M.; Sibeoni, J.; Basheer, S.; et al. Global child and adolescent mental health perspectives: bringing change locally, while thinking globally. *Child and Adolescent Psychiatry and Mental Health* **2022**, *16*, doi:10.1186/s13034-022-00512-8.

11. Gordon, B.R.; McDowell, C.P.; Hallgren, M.; et al. Association of Efficacy of Resistance Exercise Training With Depressive Symptoms Meta-analysis and Meta-regression Analysis of Randomized Clinical Trials. *Jama Psychiatry* **2018**, *75*, 566-576, doi:10.1001/jamapsychiatry.2018.0572.

12. Gilbert, A.S.; Schmidt, L.; Beck, A.; et al. Associations of physical activity and sedentary behaviors with child mental well-being during the COVID-19 pandemic. *BMC Public Health* **2021**, *21*, 1770, doi:10.1186/s12889-021-11805-6.

13. Fergusson, D.M.; Horwood, L.J.; Ridder, E.M.; et al. Subthreshold depression in adolescence and mental health outcomes in adulthood. *Archives of general psychiatry* **2005**, *62*, 66-72, doi:10.1001/archpsyc.62.1.66.

14. Brunoni, A.R.; Chaimani, A.; Moffa, A.H.; et al. Repetitive transcranial magnetic stimulation for the acute treatment of major depressive episodes: a systematic review with network meta-analysis. **2017**, *74*, 143-152.

15. Baena-Extremera, A.; Granero-Gallegos, A.; Ponce-de-Leon-Elizondo, A.; et al. Psychological factors related to physical education classes as predictors of students' intention to partake in leisure-time physical activity. *Ciencia & Saude Coletiva* **2016**, *21*, 1105-1112, doi:10.1590/1413-81232015214.07742015.

16. Ahlen, J.; Lenhard, F.; Ghaderi, A.J.T.j.o.p.p. Universal prevention for anxiety and depressive symptoms in children: A meta-analysis of randomized and cluster-randomized trials. **2015**, *36*, 387-403.

17. Hetrick, S.E.; McKenzie, J.E.; Cox, G.R.; et al. Newer generation antidepressants for depressive disorders in children and adolescents. *Cochrane Database of Systematic Reviews* **2012**, doi:10.1002/14651858.CD004851.pub3.

18. Zhao, J.L.; Jiang, W.T.; Wang, X.; et al. Exercise, brain plasticity, and depression. *CNS neuroscience & therapeutics* **2020**, *26*, 885-895, doi:10.1111/cns.13385.

19. Xu, Z.; Sun, W.; Zhang, D.; et al. Comparative effectiveness of non-pharmacological interventions for depressive symptoms in mild cognitive impairment: systematic review with network meta-analysis. *Aging & Mental Health* **2022**, *26*, 2129-2135, doi:10.1080/13607863.2021.1998356.

20. Werner-Seidler, A.; Perry, Y.; Calear, A.L.; et al. School-based depression and anxiety prevention programs for young people: A systematic review and meta-analysis. **2017**, *51*, 30-47.

21. Wang, M.; Tao, F.B.; Wu, X.Y. Research progress on the comorbidity of anxiety and depression in children and adolescents. *Chinese journal of preventive medicine* **2022**, *56*, 1011-1016, doi:10.3760/cma.j.cn112150-20220325-00283.

22. Stanton, R.; Reaburn, P. Exercise and the treatment of depression: a review of the exercise program variables. *science and medicine in sport* **2014**, *17*, 177-182.

23. Song, J.; Liu, Z.-z.; Huang, J.; et al. Effects of aerobic exercise, traditional Chinese exercises, and meditation on depressive symptoms of college student A meta-analysis of randomized controlled trials. *Medicine* **2021**, *100*, doi:10.1097/md.0000000000023819.

24. Sallis, J.F.; Prochaska, J.J.; Taylor, W.C. A review of correlates of physical activity of children and adolescents. *Medicine and science in sports and exercise* **2000**, *32*, 963-975, doi:10.1097/00005768-200009000-00012.

25. Ruotsalainen, H.; Kyng?S, H.; Tammelin, T.; et al. Systematic review of physical activity and exercise interventions on body mass indices, subsequent physical activity and psychological symptoms in overweight and obese adolescents. **2015**, *71*, 2461-2477.

26. Rodriguez-Ayllon, M.; Cadenas-Sánchez, C.; Estévez-López, F.; et al. Role of physical activity and sedentary behavior in the mental health of preschoolers, children and adolescents: a systematic review and meta-analysis. **2019**, *49*, 1383-1410.

27. Recchia, F.; Leung, C.K.; Chin, E.C.; et al. Comparative effectiveness of exercise, antidepressants and their combination in treating non-severe depression: a systematic review and network meta-analysis of randomised controlled trials. *British Journal of Sports Medicine* **2022**, *56*, 1375-+, doi:10.1136/bjsports-2022-105964.

28. Rajkumar, R.P.J.A.j.o.p. COVID-19 and mental health: A review of the existing literature. **2020**, *52*, 102066.

29. Oberste, M.; Medele, M.; Javelle, F.; et al. Physical Activity for the Treatment of Adolescent Depression: A Systematic Review and Meta-Analysis. *Frontiers in Physiology* **2020**, *11*, doi:10.3389/fphys.2020.00185.

30. Melton, T.H.; Croarkin, P.E.; Strawn, J.R.; et al. Comorbid Anxiety and Depressive Symptoms in Children and Adolescents: A Systematic Review and Analysis. *Journal of Psychiatric Practice* **2016**, *22*, 84-98, doi:10.1097/pra.0000000000000132.

31. Maughan, B.; Collishaw, S.; Stringaris, A. Depression in childhood and adolescence. *Journal of the Canadian Academy of Child and Adolescent Psychiatry = Journal de l'Academie canadienne de psychiatrie de l'enfant et de l'adolescent* **2013**, *22*, 35-40.

32. MacQueen, G.M.; Frey, B.N.; Ismail, Z.; et al. Canadian Network for Mood and Anxiety Treatments (CANMAT) 2016 Clinical Guidelines for the Management of Adults with Major Depressive Disorder: Section 6. Special Populations: Youth, Women, and the Elderly. *Canadian Journal of Psychiatry-Revue Canadienne De Psychiatrie* **2016**, *61*, 588-603, doi:10.1177/0706743716659276.

33. Kvam, S.; Kleppe, C.L.; Nordhus, I.H.; et al. Exercise as a treatment for depression: A meta-analysis. *Journal of Affective Disorders* **2016**, *202*, 67-86, doi:10.1016/j.jad.2016.03.063.

34. Kennedy, S.H. Core symptoms of major depressive disorder: relevance to diagnosis and treatment. *Dialogues in clinical neuroscience* **2008**, *10*, 271-277.

35. Kandola, A.; Ashdown-Franks, G.; Hendrikse, J.; et al. Physical activity and depression: Towards understanding the antidepressant mechanisms of physical activity. *Neuroscience and Biobehavioral Reviews* **2019**, *107*, 525-539, doi:10.1016/j.neubiorev.2019.09.040.

36. Josefsson, T.; Lindwall, M.; Archer, T. Physical exercise intervention in depressive disorders: Meta- analysis and systematic review. *Scandinavian Journal of Medicine & Science in Sports* **2014**, *24*, 259-272, doi:10.1111/sms.12050.

37. Johnstone, K.M.; Kemps, E.; Chen, J.W. A Meta-Analysis of Universal School-Based Prevention Programs for Anxiety and Depression in Children. *Clinical Child and Family Psychology Review* **2018**, *21*, 466-481, doi:10.1007/s10567-018-0266-5.

38. James-Palmer, A.; Anderson, E.Z.; Zucker, L.; et al. Yoga as an Intervention for the Reduction of Symptoms of Anxiety and Depression in Children and Adolescents: A Systematic Review. *Frontiers in Pediatrics* **2020**, *8*, doi:10.3389/fped.2020.00078.

39. Hoare, E.; Milton, K.; Foster, C.; et al. The associations between sedentary behaviour and mental health among adolescents: a systematic review. *International Journal of Behavioral Nutrition and Physical Activity* **2016**, *13*, doi:10.1186/s12966-016-0432-4.

40. 이위환; 전순한; 김용주. The Effect of Children’s Physical Activity to decrease children’s stress. *The Korean Journal Child Education* **2008**, *17*, 267-274.

41. 변정은; 이필영; 스포츠사이언스, 김.J. 초등학생들의 신체활동수준이 스트레스, 우울 및 자아존중감에 미치는 영향. **2019**, *37*, 117-126.

42. Sun, Y.-L.; Wang, J.; Yao, J.-X.; et al. Physical exercise and mental health: cognition, anxiety, depression and self-concept. **2014**, *45*, 337-342.

43. So, W.; Choi, D.; Yoon, Y.J.M.i.T.T.K.J.H.P. Effects of 14-week Circuit Weight Training on Body Composition, Cardiorespiratory Function, Fitness, Physical Self-Efficacy Scale, Beck Depression Inventory, and SF-36. **2009**, *9*, 321-328.

44. Pantoja Vallejo, A.; Polanco Zuleta, K.M.J.R.I.d.D.y.E.-e.A.P. Depression, Anxiety and Physical Activity in School Children: Comparative Study. **2019**, *3*, 143-155.

45. Niederer, D.; Vogt, L.; Staschke, V.; et al. Activity trails in the therapy of clinical depression: A randomized controlled equivalence trial/Bewegungsparcours in der Therapie klinischer Depression--eine randomisiert-kontrollierte Aquivalenzstudie. **2017**, *63*, 163-176.

46. Lu, X.; Wang, D.; Yu, D.J.R.A.d.C.P. EFFECT OF SOLUTION-FOCUSED BRIEF THERAPY-BASED ON EXERCISE PRESCRIPTION INTERVENTION ON ADOLESCENT MENTAL HEALTH/EFECTO DE LA INTERVENCIÓN DE PRESCRIPCIÓN DE EJERCICIO BASADA EN LA TERAPIA BREVE CENTRADA EN SOLUCIONES EN LA SALUD MENTAL DE LOS ADOLESCENTES. **2017**, *26*, 347.

47. López-Rodríguez, M.M.; Fernandez-Martinez, M.; Mataran-Penarrocha, G.A.; et al. Effectiveness of aquatic biodance on sleep quality, anxiety and other symptoms in patients with fibromyalgia. **2012**, *141*, 471-478.

48. Knechtle, B.J.P. Influence of physical activity on mental well-being and psychiatric disorders. **2004**, *93*, 1403-1411.

49. Choi, S.-J. Effect of an 8-Week Competitive Exercise Program on Physical Fitness and Psychological Factors in Game-addicted Adolescent. **2017**.

50. Neville, R.D.; Nelson, M.A.; Madigan, S.; et al. Does physical activity moderate the association between screen time and psychosocial development in early childhood? Analysis of a longitudinal infant cohort study in Ireland. *European journal of pediatrics* **2021**, *180*, 2199-2211, doi:10.1007/s00431-021-04008-z.

51. Lawrence, D.; Johnson, S.; Hafekost, J.; et al. The mental health of children and adolescents: Report on the second Australian child and adolescent survey of mental health and wellbeing. **2015**.

52. Wang, W.; Tang, X.J.C.S. Clinical study on aromatherapy combined with physical exercise in treating insomnia symptoms of adolescent depression. **2023**, *28*, S26-S26.

53. Osborn, T.L.; Wasil, A.R.; Venturo-Conerly, K.E.; et al. Group intervention for adolescent anxiety and depression: outcomes of a randomized trial with adolescents in Kenya. **2020**, *51*, 601-615.

54. Xie, Y.; Wu, Z.; Sun, L.; et al. The effects and mechanisms of exercise on the treatment of depression. **2021**, *12*, 705559.

55. Weersing, V.R.; Brent, D.A.; Rozenman, M.S.; et al. Brief Behavioral Therapy for Pediatric Anxiety and Depression in Primary Care A Randomized Clinical Trial. *Jama Psychiatry* **2017**, *74*, 571-578, doi:10.1001/jamapsychiatry.2017.0429.

56. Schmaal, L.; Veltman, D.J.; van Erp, T.G.; et al. Subcortical brain alterations in major depressive disorder: findings from the ENIGMA Major Depressive Disorder working group. **2016**, *21*, 806-812.

57. Salazar, G.; Vasquez, F.; Concha, F.; et al. Pilot nutrition and physical activity intervention for preschool children attending daycare centres (JUNJI): primary and secondary outcomes. *Nutricion hospitalaria* **2014**, *29*, 1004-1012, doi:10.3305/nh.2014.29.5.7316.

58. Ranney, R.M.; Behar, E.; Zinsser, K.M. Gender as a Moderator of the Relationship Between Parental Anxiety and Adolescent Anxiety and Depression. *Journal of Child and Family Studies* **2021**, *30*, 1247-1260, doi:10.1007/s10826-021-01931-5.

59. Pophillat, E.; Rooney, R.M.; Nesa, M.; et al. Preventing Internalizing Problems in 6-8 Year Old Children: A Universal School-Based Program. *Frontiers in Psychology* **2016**, *7*, doi:10.3389/fpsyg.2016.01928.

60. Pojednic, R.; Peabody, S.; Carson, S.; et al. The effect of before school physical activity on child development: A study protocol to evaluate the Build Our Kids Success (BOKS) Program. *Contemporary clinical trials* **2016**, *49*, 103-108, doi:10.1016/j.cct.2016.06.009.

61. Johnson, W.; Mortensen, E.L.; Kyvik, K.O. Gene-Environment Interplay Between Physical Exercise and Fitness and Depression Symptomatology. *Behavior genetics* **2020**, *50*, 346-362, doi:10.1007/s10519-020-10009-9.

62. Drieberg, H.; McEvoy, P.M.; Hoiles, K.J.; et al. An examination of direct, indirect and reciprocal relationships between perfectionism, eating disorder symptoms, anxiety, and depression in children and adolescents with eating disorders. *Eating Behaviors* **2019**, *32*, 53-59, doi:10.1016/j.eatbeh.2018.12.002.

63. Downs, D.S.; Savage, J.S.; DiNallo, J.M. Self-Determined to Exercise? Leisure-Time Exercise Behavior, Exercise Motivation, and Exercise Dependence in Youth. *Journal of Physical Activity & Health* **2013**, *10*, 176-184, doi:10.1123/jpah.10.2.176.

64. Bodicherla, K.P.; Shah, K.; Singh, R.; et al. School-Based Approaches to Prevent Depression in Adolescents. *Cureus* **2021**, *13*, e13443-e13443, doi:10.7759/cureus.13443.

65. Young, C.C.; Dietrich, M.S.J.J.o.C.; Nursing, A.P. Stressful life events, worry, and rumination predict depressive and anxiety symptoms in young adolescents. **2015**, *28*, 35-42.

66. Wieber, F.; Zysset, A.; al., e. Central concepts in mental health promotion programs for children and adolescents: Evidence and examples from Switzerland. *European Journal of Public Health* **2022**, Supplement_3.

67. Wang, M.; Tao, F.; al., e. Research progress on the comorbidity of anxiety and depression in children and adolescents. *Zhonghua yu fang yi xue za zhi* **2022**, *56*, 1011-1016.

68. Tiner, S.; Cunningham, G.B.; Pittman, A.J.A. “Physical activity is beneficial to anyone, including those with ASD”: Antecedents of nurses recommending physical activity for people with autism spectrum disorder. **2021**, *25*, 576-587.

69. Seok-Min, Y.; 김도호. Effects of Game & Physical Activity Program on Emotional Behaviors of Children with Pervasive Developmental Disorder in Pre-school. *Journal of adapted physical activity and exercise* **2008**, *16*, 1-23.

70. Song, J.; Liu, Z.-z.; Huang, J.; et al. Effects of aerobic exercise, traditional Chinese exercises, and meditation on depressive symptoms of college student: A meta-analysis of randomized controlled trials. **2021**, *100*.

71. Rooney, R.M.; Morrison, D.; Hassan, S.; et al. Prevention of internalizing disorders in 9-10 year old children: efficacy of the Aussie Optimism Positive Thinking Skills Program at 30-month follow-up. *Frontiers in Psychology* **2013**, *4*, doi:10.3389/fpsyg.2013.00988.

72. Kim, S.-Y.; Jeon, S.-W.; Shin, D.-W.; et al. Association between physical activity and depressive symptoms in general adult populations: An analysis of the dose-response relationship. *Psychiatry Research* **2018**, *269*, 258-263, doi:10.1016/j.psychres.2018.08.076.

73. Kekäläinen, T.; Kokko, K.; Sipilä, S.; et al. Effects of a 9-month resistance training intervention on quality of life, sense of coherence, and depressive symptoms in older adults: randomized controlled trial. **2018**, *27*, 455-465.

74. Kanning, M.; Do, B.; Mason, T.B.; et al. Doing exercise or sport together with one's child is positively associated with mothers' momentary affect in daily life, but not with higher levels of overall physical activity. *BMC Public Health* **2020**, *20*, 715, doi:10.1186/s12889-020-08864-6.

75. Gordon, B.A.; Knapman, L.M.; Lubitz, L. Graduated exercise training and progressive resistance training in adolescents with chronic fatigue syndrome: a randomized controlled pilot study. *Clinical Rehabilitation* **2010**, *24*, 1072-1079, doi:10.1177/0269215510371429.

76. Chang, Y.-C.; Lu, M.-C.; Hu, I.-H.; et al. Effects of different amounts of exercise on preventing depressive symptoms in community-dwelling older adults: a prospective cohort study in Taiwan. **2017**, *7*, e014256.

77. Callaghan, P.; Khalil, E.; Morres, I.; et al. Pragmatic randomised controlled trial of preferred intensity exercise in women living with depression. *Bmc Public Health* **2011**, *11*, doi:10.1186/1471-2458-11-465.

78. de Bruin, E.I.; van der Zwan, J.E.; Bogels, S.M. A RCT Comparing Daily Mindfulness Meditations, Biofeedback Exercises, and Daily Physical Exercise on Attention Control, Executive Functioning, Mindful Awareness, Self-Compassion, and Worrying in Stressed Young Adults. *Mindfulness* **2016**, *7*, 1182-1192, doi:10.1007/s12671-016-0561-5.

79. Cocca, A.; Espino Verdugo, F.; Rodenas Cuenca, L.T.; et al. Effect of a Game-Based Physical Education Program on Physical Fitness and Mental Health in Elementary School Children. *International Journal of Environmental Research and Public Health* **2020**, *17*, doi:10.3390/ijerph17134883.

80. Essau, C.A.; Conradt, J.; Sasagawa, S.; et al. Prevention of Anxiety Symptoms in Children: Results From a Universal School-Based Trial. *Behavior Therapy* **2012**, *43*, 450-464, doi:10.1016/j.beth.2011.08.003.

81. Williamson, D.; Dewey, A.; Steinberg, H. Mood change through physical exercise in nine- to ten-year-old children. *Perceptual and motor skills* **2001**, *93*, 311-316, doi:10.2466/pms.93.5.311-316.

82. Wilczyńska, D.; Walczak-Kozłowska, T.; Alarcón, D.; et al. Dimensions of Athlete-Coach Relationship and Sport Anxiety as Predictors of the Changes in Psychomotor and Motivational Welfare of Child Athletes after the Implementation of the Psychological Workshops for Coaches. *Int J Environ Res Public Health* **2022**, *19*, doi:10.3390/ijerph19063462.

83. Whitney, D.G.; Shapiro, D.N.; Warschausky, S.A.; et al. The contribution of neurologic disorders to the national prevalence of depression and anxiety problems among children and adolescents. *Annals of Epidemiology* **2019**, *29*, 81-84, doi:10.1016/j.annepidem.2018.11.003.

84. Vani, M.F.; Pila, E.; deJonge, M.; et al. 'Can you move your fat ass off the baseline?' Exploring the sport experiences of adolescent girls with body image concerns. *Qualitative Research in Sport Exercise and Health* **2021**, *13*, 671-689, doi:10.1080/2159676x.2020.1771409.

85. Tercedor, P.; Villa-Gonzalez, E.; Avila-Garcia, M.; et al. A school-based physical activity promotion intervention in children: rationale and study protocol for the PREVIENE Project. *Bmc Public Health* **2017**, *17*, doi:10.1186/s12889-017-4788-4.

86. Tao, R.; Liang, S.; Bao, C.; et al. Relationships Between Physical Activity, Sedentary Behavior and Anxiety in Chinese Children with Visual Impairment: A Cross-lagged Analysis. *Journal of Developmental and Physical Disabilities* **2022**, doi:10.1007/s10882-022-09879-0.

87. Soltani Kouhbanani, S.; Rothenberger, A. Perceptual-Motor Skills Reconstruction Program Improves Executive Functions in Children with Attention-Deficit/Hyperactivity Disorder. *Sustainability* **2021**, *13*, doi:10.3390/su13116210.

88. Shomaker, L.B.; Pivarunas, B.; Annameier, S.K.; et al. One-Year Follow-Up of a Randomized Controlled Trial Piloting a Mindfulness-Based Group Intervention for Adolescent Insulin Resistance. *Front Psychol* **2019**, *10*, 1040, doi:10.3389/fpsyg.2019.01040.

89. Salaj, S.; Masnjak, M. Correlation of Motor Competence and Social-Emotional Wellbeing in Preschool Children. *Frontiers in Psychology* **2022**, *13*, doi:10.3389/fpsyg.2022.846520.

90. Ridgers, N.D.; Graves, L.E.; Foweather, L.; et al. Examining influences on boy's and girls' physical activity patterns: the A-CLASS project. *Pediatric exercise science* **2010**, *22*, 638-650, doi:10.1123/pes.22.4.638.

91. Min, K.; 이종민. Influence of Sport Participation of Elementary School Children on Emotion, Self-regulation and Adaption of School. *The Korean Journal of Elementary Physical Education* **2015**, *20*, 147-159.

92. Bohnert, A.M.; Ward, A.K. Making a Difference: Evaluating the Girls in the Game (GIG) After-School Program. *Journal of Early Adolescence* **2013**, *33*, 104-130, doi:10.1177/0272431612466174.

93. Bazzano, A.N.; Anderson, C.E.; Hylton, C.; et al. Effect of mindfulness and yoga on quality of life for elementary school students and teachers: results of a randomized controlled school-based study. *Psychology Research and Behavior Management* **2018**, *11*, 81-89, doi:10.2147/prbm.S157503.

94. Battaglia, G.; Alesi, M.; Tabacchi, G.; et al. The Development of Motor and Pre-literacy Skills by a Physical Education Program in Preschool Children: A Non-randomized Pilot Trial. *Front Psychol* **2018**, *9*, 2694, doi:10.3389/fpsyg.2018.02694.

95. Barrett, P.; Turner, C. Prevention of anxiety symptoms in primary school children: preliminary results from a universal school-based trial. *The British journal of clinical psychology* **2001**, *40*, 399-410, doi:10.1348/014466501163887.

96. Wang, C. The role of physical activity promoting thinking skills and emotional behavior of preschool children. *Psicologia, reflexao e critica : revista semestral do Departamento de Psicologia da UFRGS* **2022**, *35*, 24, doi:10.1186/s41155-022-00223-1.

97. Telles, S.; Singh, N.; Bhardwaj, A.K.; et al. Effect of yoga or physical exercise on physical, cognitive and emotional measures in children: a randomized controlled trial. *Child and Adolescent Psychiatry and Mental Health* **2013**, *7*, doi:10.1186/1753-2000-7-37.

98. Stella, S.G.; Vilar, A.P.; Lacroix, C.; et al. Effects of type of physical exercise and leisure activities on the depression scores of obese Brazilian adolescent girls. *Brazilian journal of medical and biological research = Revista brasileira de pesquisas medicas e biologicas* **2005**, *38*, 1683-1689, doi:10.1590/s0100-879x2005001100017.

99. Standage, M.; Cumming, S.P.; Gillison, F.B. A cluster randomized controlled trial of the be the best you can be intervention: effects on the psychological and physical well-being of school children. *Bmc Public Health* **2013**, *13*, doi:10.1186/1471-2458-13-666.

100. Shannon, S.; Brennan, D.; Hanna, D.; et al. The Effect of a School-Based Intervention on Physical Activity and Well-Being: a Non-Randomised Controlled Trial with Children of Low Socio-Economic Status. *Sports Medicine-Open* **2018**, *4*, doi:10.1186/s40798-018-0129-0.

101. Roberts, C.M.; Kane, R.; Bishop, B.; et al. The prevention of anxiety and depression in children from disadvantaged schools. *Behaviour Research and Therapy* **2010**, *48*, 68-73, doi:10.1016/j.brat.2009.09.002.

102. Ren, Y.; Li, M. Influence of physical exercise on social anxiety of left-behind children in rural areas in China: The mediator and moderator role of perceived social support. *Journal of Affective Disorders* **2020**, *266*, 223-229, doi:10.1016/j.jad.2020.01.152.

103. Rasing, S.P.A.; Braam, M.W.G.; Brunwasser, S.M.; et al. Depression and Anxiety Symptoms in Female Adolescents: Relations with Parental Psychopathology and Parenting Behavior. *Journal of Research on Adolescence* **2020**, *30*, 298-313, doi:10.1111/jora.12525.

104. Piek, J.P.; Bradbury, G.S.; Elsley, S.C.; et al. Motor Coordination and Social-Emotional Behaviour in Preschool-aged Children. *International Journal of Disability Development and Education* **2008**, *55*, 143-151, doi:10.1080/10349120802033592.

105. Olive, L.S.; Sciberras, E.; Berkowitz, T.S.; et al. Child and Parent Physical Activity, Sleep, and Screen Time During COVID-19 and Associations With Mental Health: Implications for Future Psycho-Cardiological Disease? *Frontiers in psychiatry* **2021**, *12*, 774858, doi:10.3389/fpsyt.2021.774858.

106. Knubben, K.; Reischies, F.M.; Adli, M.; et al. A randomised, controlled study on the effects of a short-term endurance training programme in patients with major depression. *British journal of sports medicine* **2007**, *41*, 29-33.

107. Knapen, J.; Vancampfort, D.; Morien, Y.; et al. Exercise therapy improves both mental and physical health in patients with major depression. *Disability and Rehabilitation* **2015**, *37*, 1490-1495, doi:10.3109/09638288.2014.972579.

108. Kall, L.B.; Malmgren, H.; Olsson, E.; et al. Effects of a Curricular Physical Activity Intervention on Children's School Performance, Wellness, and Brain Development. *Journal of School Health* **2015**, *85*, 704-713, doi:10.1111/josh.12303.

109. Jouper, J.; Hassmen, P. Exercise intention, age and stress predict increased qigong exercise adherence. *Journal of bodywork and movement therapies* **2009**, *13*, 205-211, doi:10.1016/j.jbmt.2008.08.002.

110. Annesi, J.J. Correlations of depression and total mood disturbance with physical activity and self-concept in preadolescents enrolled in an after-school exercise program. *Psychological reports* **2005**, *96*, 891-898, doi:10.2466/pr0.96.4.891-898.
