# Supplementary material for: Effect of exercise intervention on depression in children and adolescents: a systematic review and network meta-analysis
Source: BMC Public Health. 2023 Oct 4;23:1918. doi: 10.1186/s12889-023-16824-z (PMC10552327; doi:10.1186/s12889-023-16824-z)
Supplement: Supplementary file 5 — Additional file 5: Outcome measurement [file 12889_2023_16824_MOESM5_ESM.docx]

**Additional file 5- Outcome measurement**

| **Author / Year** | **Tool** | **Tool Description** |
| --- | --- | --- |
| Annesi 2005[1] | The Profile of Mood States-Short Form scales | The Profile of Mood States-Short Form scales of Depression (5 items) and Total Mood Disturbance (30 items) |
| Bonhauser 2005[2] | The hospital anxiety depression scale (HADS) | HADS is to estimate anxiety and depressive symptoms. |
| Brown 1992[3] | The Beck Depression Inventory | The BDI was developed by Dr. Aaron T. Beck, a psychiatrist, and released in 1961. The BDI includes 21 items, each of which corresponds to a symptom of depression. |
| Butzer 2016[4] | The Brunel University Mood Scale (BRUMS) | The BRUMS was designed to assess mood in adolescents, which contains 24 adjectives that are rated on a four-point scale to give a total mood score and scores for six subscales: tension, depression, anger, vigor, fatigue, and confusion. |
| Carter 2015[5] | The Children’s depression inventory 2(CDI-2) | The CDI-2 is a 28-item self-report questionnaire designed to assess the severity of current/recent depressive symptoms in adolescents aged 7 to 17. |
| Costigan 2016[6] | The Kessler Psychological Distress Scale (K10) | The Kessler Psychological Distress Scale (K10) involves 10 questions about a person's emotional state, which is intended to measure distress based on questions about anxiety and depressive symptoms that a person has experienced in the last 4-weeks. |
| Crews 2004[7] | The Beck Depression Inventory | The BDI was developed by Dr. Aaron T. Beck, a psychiatrist, and released in 1961. The BDI includes 21 items, each of which corresponds to a symptom of depression. |
| Daley 2006[8] | The Children’s Depression Inventory (CDI) | The CDI is a 27-item, self-rated, symptom-orientated scale suitable for school-aged youngsters and adolescents. The following subscales are included: negative mood, interpersonal problems, ineffectiveness, anhedonia, and negative self-esteem. |
| Essau 2012[9] | The Revised Child Anxiety and Depression Scale (RCADS） | RCADS is consists of 11 items that correspond to the nine symptoms of Criterion A for major depressive episode. |
| Goldfield 2015[10] | The 24-item Brunell Mood Scale (BRUMS) | BRUMS has six subscales; Fatigue, Anger, Tension, Confusion, Vigor, and Depression, with each subscale consisting of four items. |
| Hilyer 1982[11] | The State-Trait Anxiety Inventory for Children and the Beck Inventory of Depression | The State-Trait Anxiety Inventory for Children measures two distinct anxiety concepts: state anxiety and trait anxiety, and the Beck Inventory of Depression is a clinically derived self-report inventory constructed to assess the current depth of depression. |
| Hughes 2013[12] | The Children’s Depression Rating Scale - Revised [CDRS-R] | CDRS-R is a clinician-rated instrument, modeled after the Hamilton Depression Rating Scale for adults, and used to measure the presence and severity of depressive symptomatology in children and adolescents. |
| Jeong 2005[13] | The Symptom Check List-90-Revision (SCL-90-R) | The clinical profiles of the SCL-90-R include dimensions of somatization (SOM), obsessive compulsive (O–C), interpersonal sensitivity (I–S), depression (DEP), anxiety (ANX), hostility (HOS), phobic anxiety (PHOB), paranoid ideation (PAR), and psychoticism(PSY). |
| Khalsa 2012[14] | The Profile of Mood States short form (POMS-SF) | The POMS-SF is a shortened version of the POMS and provides a total mood disturbance score as well as subscale scores for six mood states. |
| Lin 2020[15] | The Bipolar Prodrome Scale-Retrospective: Patient Version (BPSS-R-Pt) | BPSS-R-Pt is to assess current and pass symptoms respectively, which is a self-reported, 74-item symptom checklist and a Chinese version of the Bipolar Prodrome Scale-Retrospective. |
| MacMahon 1988[16] | The Beck Depression Inventory | The BDI was developed by Dr. Aaron T. Beck, a psychiatrist, and released in 1961. The BDI includes 21 items, each of which corresponds to a symptom of depression. |
| Mohammadi 2011[17] | The Beck Depression Inventory | The Beck Depression Inventory contains 21 questions which altogether assesses 21 aspects of depression. |
| Nabkasorn 2006[18] | Epidemiologic Studies Depression (CES-D) rating scale | The CES-D is a self-reported measure of the frequency of 20 depressive symptoms during the past week. |
| Norris 1992[19] | The Multiple Affect Adjective Check List | Respondents are asked to indicate which of 132 adjectives describe the way they generally feel. For each scale; Anxiety, Depression and Hostility there are a number of adjectives which are given a plus or minus rating. The number of plus items which are ticked are scored and the number of minus items which are not ticked are also scored. |
| Olive 2019[20] | The Children’s Depression Inventory (CDI) and the Children’s Stress Questionnaire (CSQ) | CDI is to assess depression, and CSQ is a 50-item inventory assessing stressor exposure and the impact of self-reported stressor experience over the past 12 months. |
| Petty 2009[21] | Reynolds Child Depression Scale (RCDS) | RCDS is a 30-item self-report Likert scale of depressive symptoms in children. |
| Roberts 2010[22] | The Child Depression Inventory (CDI） | The CDI is used to measure depressed affect, somatic symptoms, depressive behaviour, low self-esteem, and anhedonia. |
| Romero-Pérez 2020[23] | The Depression Scale in Children (CDS) | The TCDS consists of a self-report questionnaire, directed at children between 8 and 16 years old, composed of 66 statements, 48 of them of a depressive type and 18 of a positive type. |
| Roshan 2011[24] | Hamilton Rating Scale for Depression (Ham-D) | Hamilton Rating Scale for Depression (Ham-D) to assess the severity of depression. |
| Roth 1987[25] | The Beck Depression Inventory (BDI) | The BDI was developed by Dr. Aaron T. Beck, a psychiatrist, and released in 1961. The BDI includes 21 items, each of which corresponds to a symptom of depression. |
| Shachar 2016[26] | The 20-item Positive and Negative Affect Schedule (PANAS) | PANAS is a self-report checklist of affect adjectives, was designed to provide independent measures of positive and negative affect. |
| Silva 2020[27] | The Child Depression Inventory (CDI) | CDI measures depressive symptoms in children and adolescents, ranging in age from seven to seventeen. Composed of 27 items, each with three response options, each of them being a corresponding value, being summed (a = 0; b = 1 and c = 2), using cut-off point 17. |
| Talakoub 2012[28] | SCL-90-r (90-item Symptom Checklist Questionnaire) | SCL-90-r is used for affective responses, and consists of two parts; the first part includes demographic characteristics and the second and main part of the SCL-90 consists of 90 dimensions. |
| Weersing 2017[29] | The Children’s Depression Rating Scale–Revised（CDRS-R） | The Children's Depression Rating Scale--Revised (CDRS-R; Poznanski et al., 1984) is a clinician-rated instrument for the assessment of the severity of depression in children ages 6-12 years. |
| Weintraub 2008[30] | The Children’s Depression Inventory. | The 10-item Children’s Depression Inventory was used to assess depressive symptoms. |
| Williams 2019[31] | The Children’s Depression Inventory (CDI) | CDI is a 27-item self-report questionnaire designed to assess depressive symptoms in children and adolescents that have occurred within 2 weeks of completing the assessment. |
| Williamson 2001[32] | The modfied mood questionnaire | The mood questionnaire is a self-report mood measure of eight positive and eight negative adjectives. |
| Wunram 2018[33] | Depression sinventar für Kinder und Jugendliche (DIKJ) | The DIKJ is a self-report questionnaire assessing the severity of depressive symptoms in children and adolescents as of grade two, conceived on the basis of the anglosaxon children’s depression inventory. |
| Yu 2020[34] | The Social Anxiety Scale for Children (SASC) | SASC was developed to evaluate children’s feelings of social anxiety in the context of their peer relations, which consists of 10 items that assess social avoidance and distress and fear of negative evaluation, and total score ≥ 7 indicates social anxiety. |
| Zhang 2021[35] | The Hamilton Depression Scale (HAMD) | The HAMD evaluation includes 24 items, including depression, sleep disorders, guilt, language or thought retardation and despair, etc. The higher the score, the more serious the depression. |

1. Annesi, J.J. Correlations of depression and total mood disturbance with physical activity and self-concept in preadolescents enrolled in an after-school exercise program. *Psychological reports* **2005**, *96*, 891-898, doi:10.2466/pr0.96.4.891-898.

2. Bonhauser, M.; Fernandez, G.; Püschel, K.; et al. Improving physical fitness and emotional well-being in adolescents of low socioeconomic status in Chile: results of a school-based controlled trial. *Health Promotion International* **2005**, *20*, 113-122, doi:10.1093/heapro/dah603.

3. Brown, H.E.; Pearson, N.; Braithwaite, R.E.; et al. Physical activity interventions and depression in children and adolescents : a systematic review and meta-analysis. *Sports Med* **2013**, *43*, 195-206, doi:10.1007/s40279-012-0015-8.

4. Butzer, B.; LoRusso, A.; Shin, S.H.; et al. Evaluation of Yoga for Preventing Adolescent Substance Use Risk Factors in a Middle School Setting: A Preliminary Group-Randomized Controlled Trial. *Journal of Youth and Adolescence* **2017**, *46*, 603-632, doi:10.1007/s10964-016-0513-3.

5. Carter, T.; Guo, B.; Turner, D.; et al. Preferred intensity exercise for adolescents receiving treatment for depression: a pragmatic randomised controlled trial. *Bmc Psychiatry* **2015**, *15*, doi:10.1186/s12888-015-0638-z.

6. Costigan, S.A.; Eather, N.; Plotnikoff, R.C.; et al. High-Intensity Interval Training for Cognitive and Mental Health in Adolescents. *Medicine & Science in Sports & Exercise* **2016**, *48*, 1985-1993, doi:10.1249/mss.0000000000000993.

7. Crews, D.J.; Lochbaum, M.R.; Landers, D.M. Aerobic physical activity effects on psychological well-being in low-income Hispanic children. *Perceptual and motor skills* **2004**, *98*, 319-324, doi:10.2466/pms.98.1.319-324.

8. Daley, A.J.; Copeland, R.J.; Wright, N.P.; et al. Exercise therapy as a treatment for psychopathologic conditions in obese and morbidly obese adolescents: a randomized, controlled trial. *Pediatrics* **2006**, *118*, 2126-2134, doi:10.1542/peds.2006-1285.

9. Essau, C.A.; Conradt, J.; Sasagawa, S.; et al. Prevention of Anxiety Symptoms in Children: Results From a Universal School-Based Trial. *Behavior Therapy* **2012**, *43*, 450-464, doi:10.1016/j.beth.2011.08.003.

10. Goldfield, G.S.; Kenny, G.P.; Alberga, A.S.; et al. Effects of aerobic training, resistance training, or both on psychological health in adolescents with obesity: The HEARTY randomized controlled trial. *Journal of consulting and clinical psychology* **2015**, *83*, 1123-1135, doi:10.1037/ccp0000038.

11. Hilyer, J.C.; Wilson, D.G.; Dillon, C.; et al. Physical fitness training and counseling as treatment for youthful offenders. **1982**, *29*, 292.

12. Hughes, C.W.; Barnes, S.; Barnes, C.; et al. Depressed Adolescents Treated with Exercise (DATE): A pilot randomized controlled trial to test feasibility and establish preliminary effect sizes. *Mental Health and Physical Activity* **2013**, *6*, 119-131, doi:10.1016/j.mhpa.2013.06.006.

13. Jeong, Y.-J.; Hong, S.-C.; Lee, M.S.; et al. Dance movement therapy improves emotional responses and modulates neurohormones in adolescents with mild depression. *The International journal of neuroscience* **2005**, *115*, 1711-1720, doi:10.1080/00207450590958574.

14. Khalsa, S.B.S.; Hickey-Schultz, L.; Cohen, D.; et al. Evaluation of the mental health benefits of yoga in a secondary school: a preliminary randomized controlled trial. *The journal of behavioral health services & research* **2012**, *39*, 80-90, doi:10.1007/s11414-011-9249-8.

15. Lin, K.; Stubbs, B.; Zou, W.; et al. Aerobic exercise impacts the anterior cingulate cortex in adolescents with subthreshold mood syndromes: a randomized controlled trial study. *Translational Psychiatry* **2020**, *10*, doi:10.1038/s41398-020-0840-8.

16. MacMahon, J.R.; Gross, R.T.J.A.J.o.D.o.C. Physical and psychological effects of aerobic exercise in delinquent adolescent males. **1988**, *142*, 1361-1366.

17. Mohammadi, M. A study and comparison of the effect of team sports (soccer and volleyball) and individual sports (table tennis and badminton) on depression among high school students. *Aust. J. Basic Appl. Sci* **2011**, *5*, 1005-1011.

18. Nabkasorn, C.; Miyai, N.; Sootmongkol, A.; et al. Effects of physical exercise on depression, neuroendocrine stress hormones and physiological fitness in adolescent females with depressive symptoms. *European journal of public health* **2006**, *16*, 179-184.

19. Norris, R.; Carroll, D.; Cochrane, R. The effects of physical activity and exercise training on psychological stress and well-being in an adolescent population. *Journal of psychosomatic research* **1992**, *36*, 55-65, doi:10.1016/0022-3999(92)90114-h.

20. Olive, L.S.; Byrne, D.; Cunningham, R.B.; et al. Can Physical Education Improve the Mental Health of Children? The LOOK Study Cluster-Randomized Controlled Trial. *Journal of Educational Psychology* **2019**, *111*, 1331-1340, doi:10.1037/edu0000338.

21. Petty, K.H.; Davis, C.L.; Tkacz, J.; et al. Exercise effects on depressive symptoms and self-worth in overweight children: a randomized controlled trial. *Journal of pediatric psychology* **2009**, *34*, 929-939.

22. Roberts, C.M.; Kane, R.; Bishop, B.; et al. The prevention of anxiety and depression in children from disadvantaged schools. *Behaviour Research and Therapy* **2010**, *48*, 68-73, doi:10.1016/j.brat.2009.09.002.

23. Romero-Pérez, E.M.; González-Bernal, J.J.; Soto-Cámara, R.; et al. Influence of a physical exercise program in the anxiety and depression in children with obesity. *International journal of environmental research and public health* **2020**, *17*, 4655.

24. Roshan, V.D.; Pourasghar, M.; Mohammadian, Z. The efficacy of intermittent walking in water on the rate of MHPG sulfate and the severity of depression. *Iranian Journal of Psychiatry and Behavioral Sciences* **2011**, *5*, 26-31.

25. Roth, D.L.; Holmes, D.S. Influence of aerobic exercise training and relaxation training on physical and psychologic health following stressful life events. *Psychosomatic Medicine* **1987**, *49*.

26. Shachar, K.; Ronen-Rosenbaum, T.; Rosenbaum, M.; et al. Reducing child aggression through sports intervention: The role of self-control skills and emotions. *Children and Youth Services Review* **2016**, *71*, 241-249, doi:10.1016/j.childyouth.2016.11.012.

27. Da Silva, L.A.; Doyenart, R.; Salvan, P.H.; et al. Swimming training improves mental health parameters, cognition and motor coordination in children with Attention Deficit Hyperactivity Disorder. *International Journal of Environmental Health Research* **2020**, *30*, 584-592, doi:10.1080/09603123.2019.1612041.

28. Sedigheh, T.; Sakineh, G.; Marzieh, H.; et al. Impact of exercise on affective responses in female adolescents with type I diabetes. **2012**.

29. Weersing, V.R.; Brent, D.A.; Rozenman, M.S.; et al. Brief Behavioral Therapy for Pediatric Anxiety and Depression in Primary Care A Randomized Clinical Trial. *Jama Psychiatry* **2017**, *74*, 571-578, doi:10.1001/jamapsychiatry.2017.0429.

30. Weintraub, D.L.; Tirumalai, E.C.; Haydel, K.F.; et al. Team sports for overweight children: The Stanford sports to prevent obesity randomized trial (SPORT). *Archives of pediatrics & adolescent medicine* **2008**, *162*, 232-237.

31. Williams, C.F.; Bustamante, E.E.; Waller, J.L.; et al. Exercise effects on quality of life, mood, and self-worth in overweight children: the SMART randomized controlled trial. *Translational behavioral medicine* **2019**, *9*, 451-459.

32. Williamson, D.; Dewey, A.; Steinberg, H. Mood change through physical exercise in nine- to ten-year-old children. *Perceptual and motor skills* **2001**, *93*, 311-316, doi:10.2466/pms.93.5.311-316.

33. Wunram, H.L.; Hamacher, S.; Hellmich, M.; et al. Whole body vibration added to treatment as usual is effective in adolescents with depression: a partly randomized, three-armed clinical trial in inpatients. **2018**, *27*, 645-662.

34. Yu, H.-j.; Li, F.; Hu, Y.-f.; et al. Improving the metabolic and mental health of children with obesity: a school-based nutrition education and physical activity intervention in Wuhan, China. *Nutrients* **2020**, *12*, 194.

35. Zhang, J.; Ji, W. Exercise intervention improves the quality of life, anxiety, and depression of adolescent depression patients. *Int J Clin Exp Med* **2021**, *14*, 1292-1300.
