# Supplementary figures and images for: Effect of exercise intervention on depression in children and adolescents: a systematic review and network meta-analysis
Source: BMC Public Health. 2023 Oct 4;23:1918. doi: 10.1186/s12889-023-16824-z (PMC10552327; doi:10.1186/s12889-023-16824-z)

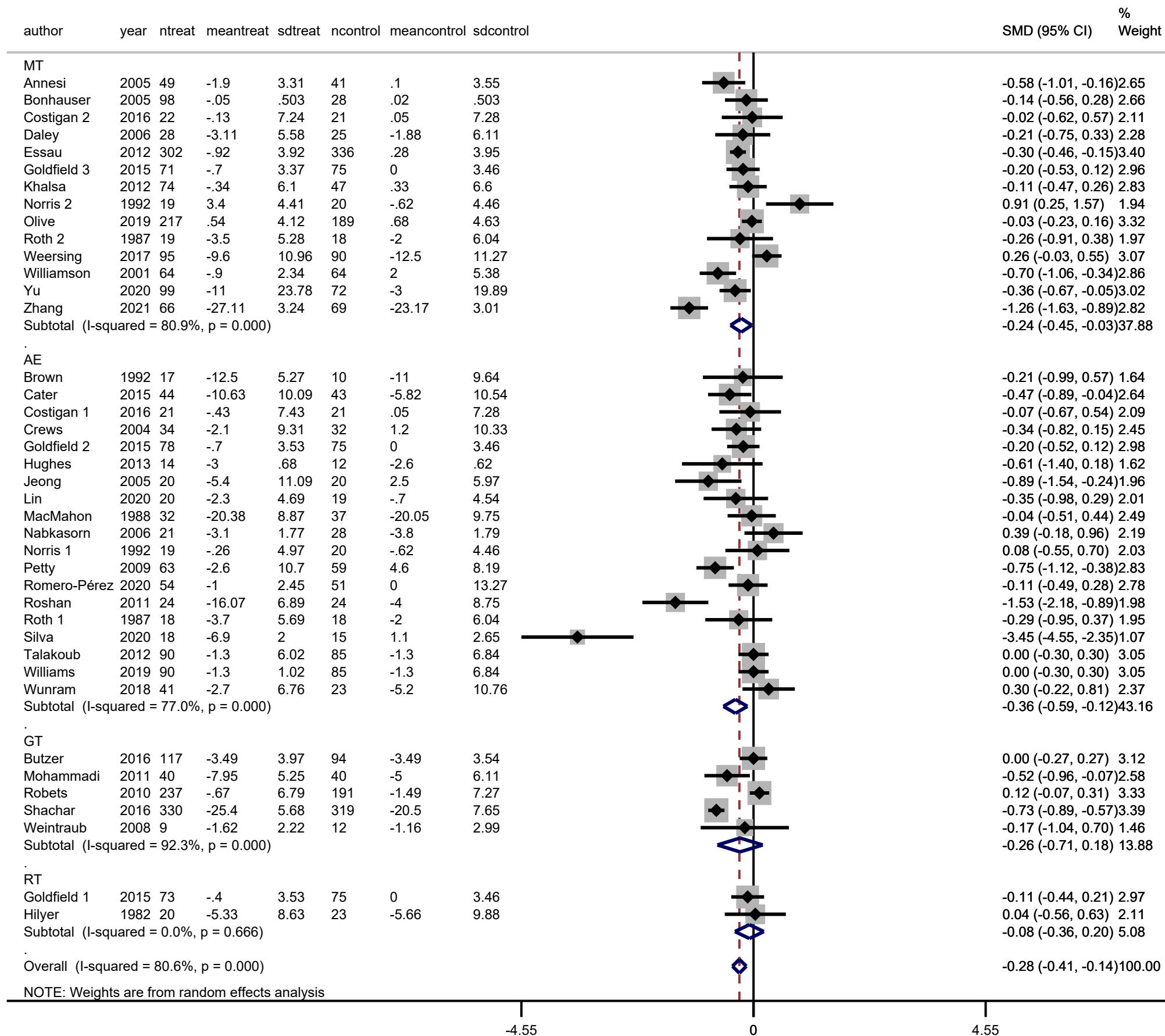

Supplement: Supplementary file 6 — Additional file 6: Forest plot [file 12889_2023_16824_MOESM6_ESM.pdf]
