# Supplementary material for: Effect of exercise intervention on depression in children and adolescents: a systematic review and network meta-analysis
Source: BMC Public Health. 2023 Oct 4;23:1918. doi: 10.1186/s12889-023-16824-z (PMC10552327; doi:10.1186/s12889-023-16824-z)
Supplement: Supplementary file 7 — Additional file 7: Risk of bias [file 12889_2023_16824_MOESM7_ESM.docx]

**Additional file 7- Risk of bias**

| Author / Year | Random sequence generation | Allocation concealment | Performance bias | Detection bias | Attrition bias | Reporting bias | Other bias |
| --- | --- | --- | --- | --- | --- | --- | --- |
| Annesi 2005[1] | H | U | H | U | L | U | U |
| Bonhauser 2005[2] | L | U | U | U | L | L | U |
| Brown 1992[3] | L | L | U | U | L | L | U |
| Butzer 2016[4] | L | U | L | U | L | L | U |
| Carter 2015[5] | L | U | U | L | U | L | U |
| Costigan 2016[6] | L | L | U | L | L | L | U |
| Crews 2004[7] | L | U | L | U | L | U | U |
| Daley 2006[8] | L | U | U | H | L | L | U |
| Essau 2012[9] | L | U | U | U | L | L | U |
| Goldfield 2015[10] | L | L | U | L | L | L | U |
| Hilyer 1982[11] | L | L | U | L | L | L | U |
| Hughes 2013[12] | L | U | U | U | L | L | U |
| Jeong 2005[13] | L | U | H | U | L | L | U |
| Khalsa 2012[14] | L | U | H | U | L | L | H |
| Lin 2020[15] | L | L | U | L | L | L | U |
| MacMahon 1988[16] | H | L | U | U | L | L | U |
| Mohammadi 2011[17] | L | L | H | U | L | L | U |
| Nabkasorn 2006[18] | L | U | L | U | L | L | U |
| Norris 1992[19] | L | L | H | L | L | L | U |
| Olive 2019[20] | L | L | U | L | L | L | U |
| Petty 2009[21] | L | L | U | U | L | L | U |
| Roberts 2010[22] | L | L | U | U | L | L | L |
| Romero-Pérez 2020[23] | L | H | U | U | U | L | U |
| Roshan 2011[24] | L | U | H | U | L | L | U |
| Roth 1987[25] | L | U | L | L | L | L | U |
| Shachar 2016[26] | H | H | U | U | L | L | U |
| Silva 2020[27] | L | U | U | U | L | L | U |
| Talakoub 2012[28] | L | U | U | U | L | L | U |
| Weersing 2017[29] | L | L | U | L | L | L | U |
| Weintraub 2008[30] | L | U | U | H | L | L | U |
| Williams 2019[31] | L | U | U | U | L | L | H |
| Williamson 2001[32] | L | U | H | U | L | U | U |
| Wunram 2018[33] | L | U | U | L | L | L | H |
| Yu 2020[34] | L | U | U | U | L | L | H |
| Zhang 2021[35] | L | L | U | U | L | L | L |

H= high risk; L= low risk; U= unclear.
