# Supplementary material for: Effect of exercise intervention on depression in children and adolescents: a systematic review and network meta-analysis
Source: BMC Public Health. 2023 Oct 4;23:1918. doi: 10.1186/s12889-023-16824-z (PMC10552327; doi:10.1186/s12889-023-16824-z)
Supplement: Supplementary file 8 — Additional file 8: Assessment of Inconsistency [file 12889_2023_16824_MOESM8_ESM.docx]

network meta i

Command is: mvmeta _y _S , bscovariance(exch 0.5) longparm suppress(uv mm) eq(_y_B: des_ABCE des_ABE, _y_D: des_DE, _y_E: des_ABE des_AE des_CE) vars(_y_B _

> y_C _y_D _y_E)

Note: using method reml

Note: regressing _y_B on des_ABCE des_ABE

Note: regressing _y_C on (nothing)

Note: regressing _y_D on des_DE

Note: regressing _y_E on des_ABE des_AE des_CE

Note: 35 observations on 4 variables

Note: variance-covariance matrix is proportional to .5*I(4)+.5*J(4,4,1)

initial: log likelihood = -55.083713

rescale: log likelihood = -55.083713

rescale eq: log likelihood = -55.083713

Iteration 0: log likelihood = -55.083713

Iteration 1: log likelihood = -54.910893

Iteration 2: log likelihood = -54.900939

Iteration 3: log likelihood = -54.900922

Iteration 4: log likelihood = -54.900922

Multivariate meta-analysis

Variance-covariance matrix = proportional .5*I(4)+.5*J(4,4,1)

Method = reml Number of dimensions = 4

Restricted log likelihood = -54.900922 Number of observations = 35

------------------------------------------------------------------------------

| Coef. Std. Err. z P>|z| [95% Conf. Interval]

-------------+----------------------------------------------------------------

_y_B |

des_ABCE | .356753 .9595657 0.37 0.710 -1.523961 2.237467

des_ABE | .3780537 .6113262 0.62 0.536 -.8201237 1.576231

_cons | -.471634 .2488265 -1.90 0.058 -.959325 .016057

-------------+----------------------------------------------------------------

_y_C |

_cons | -.2011425 .9259963 -0.22 0.828 -2.016062 1.613777

-------------+----------------------------------------------------------------

_y_D |

des_DE | .1267753 1.390012 0.09 0.927 -2.597598 2.851149

_cons | -.3276019 .4721874 -0.69 0.488 -1.253072 .5978685

-------------+----------------------------------------------------------------

_y_E |

des_ABE | .5056919 1.081961 0.47 0.640 -1.614913 2.626297

des_AE | .2370504 .9725742 0.24 0.807 -1.66916 2.143261

des_CE | 1.156256 1.342092 0.86 0.389 -1.474197 3.786708

_cons | -.2009118 .9266771 -0.22 0.828 -2.017166 1.615342

------------------------------------------------------------------------------

Estimated between-studies SDs and correlation matrix:

SD _y_B _y_C _y_D _y_E

_y_B .91223112 1 . . .

_y_C .91223112 .5 1 . .

_y_D .91223112 .5 .5 1 .

_y_E .91223112 .5 .5 .5 1

Testing for inconsistency:

( 1) [_y_B]des_ABCE = 0

( 2) [_y_B]des_ABE = 0

( 3) [_y_E]des_ABE = 0

( 4) [_y_E]des_AE = 0

( 5) [_y_E]des_CE = 0

( 6) [_y_D]des_DE = 0

chi2( 6) = 1.42

Prob > chi2 = 0.9646

mvmeta command stored as F9; test command stored as F8

.
