# Supplementary material for: Effect of exercise intervention on depression in children and adolescents: a systematic review and network meta-analysis
Source: BMC Public Health. 2023 Oct 4;23:1918. doi: 10.1186/s12889-023-16824-z (PMC10552327; doi:10.1186/s12889-023-16824-z)
Supplement: Supplementary file 9 — Additional file 9: Abbreviations [file 12889_2023_16824_MOESM9_ESM.docx]

| **Abbreviations** | **Definition** |
| --- | --- |
| WHO | World Health Organization |
| Aerobic exercise, AE | Exercise such as continuous or low-intensity running, such as walking, long-distance jogging, cycling, swimming. |
| Medium-low-intensity multi-motion training, MT | Exercises such as dance, martial arts, yoga, Pilates, aerobics, baduanjin, Tai chi or low-intensity aerobic and strength alternating combination exercises |
| Resistance training, RT | Resistance exercise by overcoming self-body mass or applying external resistance. Weight training exercises for strengthening the trunk and extremities' major muscle groups including upper and lower bodies. |
| Game training, GT | Football, basketball, badminton and other group sports and somatosensory games or game competitio |
